# Metastatic tumor growth in steatotic liver is promoted by HAS2-mediated fibrotic tumor microenvironment

**DOI:** 10.1172/JCI180802

**Published:** 2025-02-13

**Authors:** Yoon Mee Yang, Jieun Kim, Zhijun Wang, Jina Kim, So Yeon Kim, Gyu Jeong Cho, Jee Hyung Lee, Sun Myoung Kim, Takashi Tsuchiya, Michitaka Matsuda, Vijay Pandyarajan, Stephen J. Pandol, Michael S. Lewis, Alexandra Gangi, Paul W. Noble, Dianhua Jiang, Akil Merchant, Edwin M. Posadas, Neil A. Bhowmick, Shelly C. Lu, Sungyong You, Alexander M. Xu, Ekihiro Seki

**Affiliations:** 1 Karsh Division of Gastroenterology and Hepatology, Department of Medicine, Cedars-Sinai Medical Center, Los Angeles, California, USA.; 2 Department of Pharmacy,; 3 Multidimensional Genomics Research Center, and; 4 Innovative Drug Development Research Team for Intractable Diseases (BK21 plus), Kangwon National University, Chuncheon, South Korea.; 5 Division of Gastroenterology, Union Hospital, Tongji Medical College, Huazhong University of Science and Technology, Wuhan, China.; 6 Samuel Oschin Comprehensive Cancer Institute,; 7 Department of Urology,; 8 Department of Computational Biomedicine, and; 9 Department of Pathology, Cedars-Sinai Medical Center, Los Angeles, California, USA.; 10 Department of Pathology, Veterans Affairs Greater Los Angeles Health Care System, Los Angeles, California, USA.; 11 Department of Surgery,; 12 Department of Medicine and Women’s Guild Lung Institute,; 13 Division of Hematology and Cellular Therapy, Department of Medicine,; 14 Division of Medical Oncology, Department of Medicine, and; 15 Department of Biomedical Sciences, Cedars-Sinai Medical Center, Los Angeles, California, USA.; 16 Fischell Department of Bioengineering, A. James Clark School of Engineering, University of Maryland, College Park, Maryland, USA.

**Keywords:** Hepatology, Oncology, Extracellular matrix, Fibrosis, Liver cancer

## Abstract

Steatotic liver enhances liver metastasis of colorectal cancer (CRC), but this process is not fully understood. Steatotic liver induced by a high-fat diet increases cancer-associated fibroblast (CAF) infiltration and collagen and hyaluronic acid (HA) production. We investigated the role of HA synthase 2 (HAS2) in the fibrotic tumor microenvironment in steatotic liver using *Has2^ΔHSC^* mice, in which *Has2* is deleted from hepatic stellate cells. *Has2^ΔHSC^* mice had reduced steatotic liver–associated metastatic tumor growth of MC38 CRC cells, collagen and HA deposition, and CAF and M2 macrophage infiltration. We found that low–molecular weight HA activates Yes-associated protein (YAP) in cancer cells, which then releases connective tissue growth factor to further activate CAFs for HAS2 expression. Single-cell analyses revealed a link between CAF-derived HAS2 and M2 macrophages and CRC cells through CD44; these cells were associated with exhausted CD8^+^ T cells via programmed death–ligand 1 and programmed cell death protein 1 (PD-1). HA synthesis inhibitors reduced steatotic liver–associated metastasis of CRC, YAP expression, and CAF and M2 macrophage infiltration, and improved response to anti–PD-1 antibody. In conclusion, steatotic liver modulates a fibrotic tumor microenvironment to enhance metastatic cancer activity through a bidirectional regulation between CAFs and metastatic tumors, enhancing the metastatic potential of CRC in the liver.

## Abstract 2

<span>Steatotic liver disease promotes colorectal cancer liver metastasis by the enhanced pro-metastatic fibrotic microenvironment, mediated by cancer-associated fibroblasts, HAS2, and hyaluronic acid</span>

## Graphical abstract

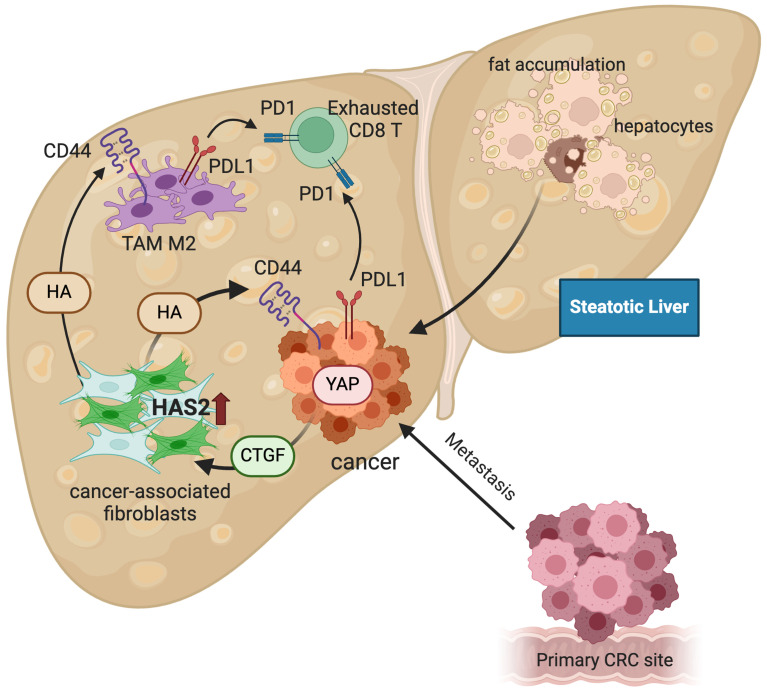

## Introduction

Colorectal cancer (CRC) metastasizes most commonly to the liver, mainly through the portal vein (1). Once liver metastasis occurs, the 5-year survival rate of patients with CRC drops from approximately 60% to 13% (2, 3). Thus, liver metastasis is a major prognostic factor in patients with CRC (1, 4). Although surgical resection is the primary treatment for CRC, only limited cases are considered for CRC resection after liver metastasis occurs (3, 5). In most cases, chemotherapy is the standard care option for patients with CRC and liver metastasis (1, 5).

Obesity is a serious health concern worldwide and is associated with the increased prevalence of metabolic dysfunction–associated steatotic liver disease (MASLD). MASLD is a common risk factor for primary liver cancer and other primary cancers, such as CRC, pancreatic cancer, prostate cancer, and breast cancer in humans and rodents (6–8). Clinical studies determined the association of obesity and MASLD with enhancement of CRC liver metastasis (9–12). Furthermore, our previous studies demonstrated the role of steatotic liver–derived extracellular vesicles and an immunosuppressive tumor microenvironment (TME) in the progression of CRC liver metastasis (13, 14). However, the underlying mechanism by which the remodeling of the fibrotic immune microenvironment by steatotic liver contributes to CRC liver metastasis is not fully understood.

Hepatic stellate cells (HSCs) are the primary source of cancer-associated fibroblasts (CAFs) that are a component of the TME in the metastatic locus in the liver (15–19). CAFs transdifferentiate into a myofibroblastic form to produce extracellular matrix (ECM), such as collagen and hyaluronic acid (HA), that modulates the prometastatic TME. The effect of ECM on cancer growth is still not fully understood. We previously determined that in liver fibrosis, HA is produced from activated HSCs through the upregulation of HA synthase 2 (HAS2) (20). We determined that low–molecular weight (LMW) HA is a dominant form in the fibrotic liver, and is proinflammatory and profibrogenic, which activates CD44, Toll-like receptor 4 (TLR4), and Notch signaling. In contrast, high–molecular weight (HMW) HA is antiinflammatory and antifibrotic (20). Likewise, the size-dependent effect of HA is observed in cancers. In breast cancer, LMW-HA activates tumor-promoting signaling through CD44 and Yes-associated protein (YAP), but HMW-HA does not (21). In the present study, we hypothesize that HSC-derived CAFs upregulate HAS2 to produce HA, which promotes CRC liver metastasis. We determined that fibrotic TME associated with HAS2 and HA is increased and plays an important role in the steatotic liver compared with the non-steatotic liver using a mouse model and patient specimens. We found that a fibrotic TME and HAS2 contribute to YAP activation in cancer cells and the development of a prometastatic immunosuppressive TME comprising M2 tumor-associated macrophages (TAMs) and exhausted CD8^+^ T cells. Lastly, we found that the drugs that inhibit HA synthesis reduced metastatic tumor burden in steatotic liver in mice, and enhanced antitumor response when added to anti–programmed cell death protein 1 (anti–PD-1) therapy. Our study provides evidence of different tumor-promoting mechanisms in patients with liver metastasis in the presence and absence of steatotic liver; we propose that disease management of patients with CRC liver metastasis should differ depending on whether or not they have steatotic liver disease.

## Results

### Increased fibrotic TME with upregulation of HAS2 and HA in CRC liver metastasis enhanced by HFD.

**Figure 1:**
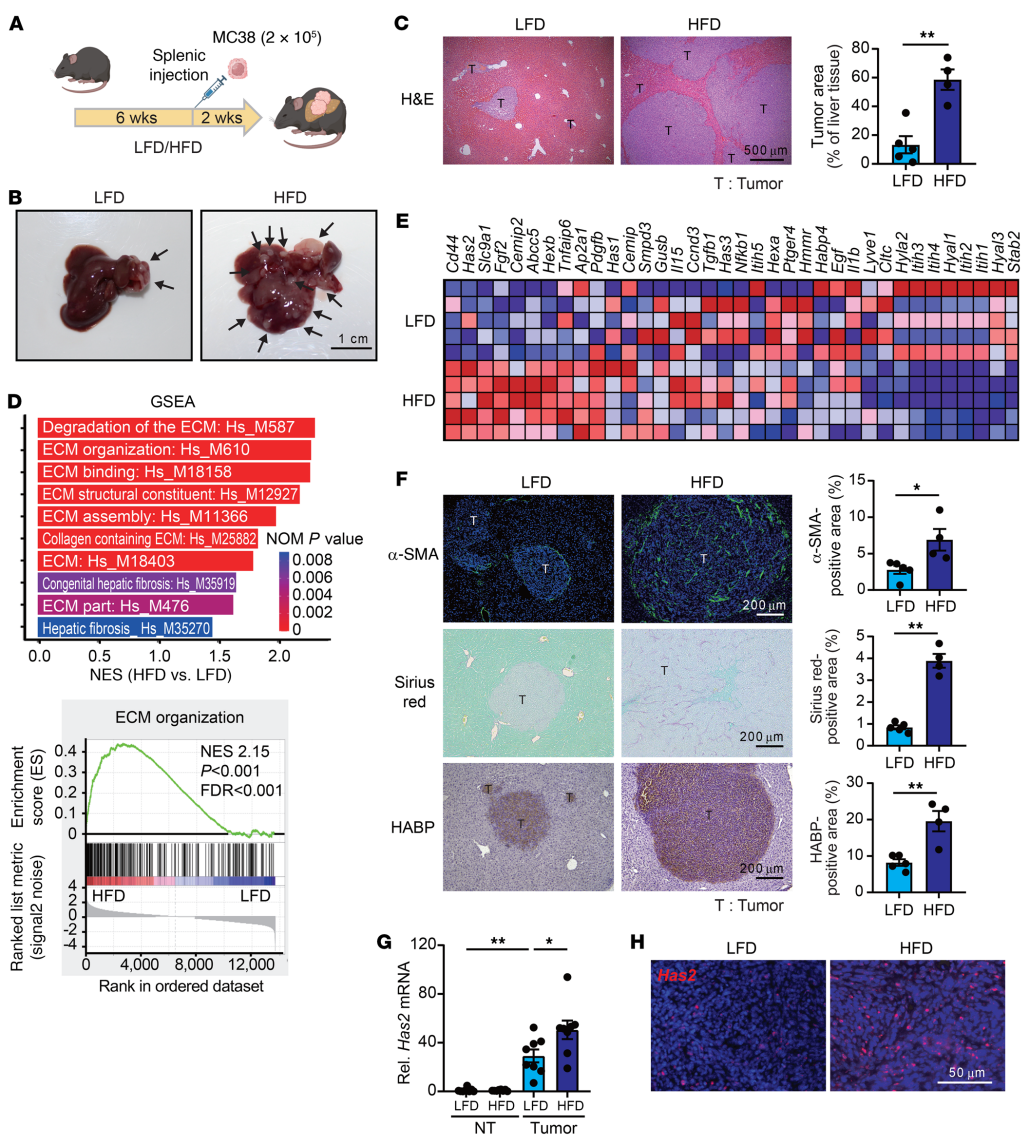
HFD-induced steatotic liver increases HA accumulation and HAS2 expression in tumors. (**A**) Representation of the mouse model illustrating the induction of steatotic liver and subsequent splenic injection of MC38 cells to form liver metastases. (**B**) Macroscopic appearance of the liver with arrows indicating tumor sites. Scale bar: 1 cm. (**C**) Representative images of hematoxylin and eosin–stained (H&E-stained) tumor and quantitative assessment of tumor area based on H&E staining. (*n* = 4–5 per group.) Scale bar: 500 μm. (**D**) RNA-Seq analysis. Top: Gene set enrichment analysis (GSEA) of gene expression for ECM and liver fibrosis in tumors from mice fed an LFD or an HFD. Bottom: Enrichment plot for ECM organization. FDR, false discovery rate; NES, normalized enrichment score; NOM, nominal. (**E**) A heatmap of the HA-related genes. (*n* = 5.) (**F**) Representative microscopic images depicting liver sections stained for α-smooth muscle actin (α-SMA), with Sirius red, and for HA-binding protein (HABP). Scale bars: 200 μm. Quantification of α-SMA–positive area, Sirius red–positive area, and HABP-positive area. (*n* = 4–5 per group.) (**G**) Comparison of mRNA expression levels of *Has2* in nontumor (NT) and tumor tissues from mice fed an LFD or an HFD. (*n* = 8 per group.) (**H**) Representative images of RNAscope in situ hybridization for *Has2*. Scale bar: 50 μm. Data are presented as mean ± SEM. Statistical significance was calculated with a 2-tailed Student’s *t* test (**C** and **F**) and 1-way ANOVA followed by Tukey’s post hoc test (**G**). *P < 0.05, **P < 0.01.

To assess the impact of steatotic liver on metastatic CRC growth in the liver, C57BL/6 male wild-type (WT) mice were fed a low-fat diet (LFD) or a high-fat diet (HFD) for 8 weeks. After the sixth week of diet feeding, MC38 cells, a murine CRC cell line, were intrasplenically injected into the mice to form liver metastases (Figure 1A) (13, 14). The area of the liver tumors was significantly larger in mice fed an HFD compared with those fed an LFD (Figure 1, B and C). RNA-Seq and gene set enrichment analysis revealed that the gene signatures of ECM organization, binding, assembly, collagen-containing ECM, and hepatic fibrosis were enriched in metastatic tumors from HFD-fed mice (Figure 1D). These data indicate that steatotic liver favors creation of a fibrotic TME. Therefore, we assessed the fibrotic TME by examining myofibroblastic CAF infiltration and deposition of ECM collagen and HA. A heatmap showed that genes associated with HA synthesis and metabolism were differentially expressed in tumors of mice fed an HFD compared with those fed an LFD (Figure 1E). Myofibroblastic CAF infiltration (as demonstrated by α-smooth muscle actin [α-SMA] expression) and collagen and HA deposition were increased in tumors of mice fed an HFD compared with those fed an LFD (Figure 1F). Next, we investigated the involvement of HA deposition in tumors. Among 3 HAS isoforms, *Has2* mRNA expression showed the greatest increase in the tumors from HFD-fed mice compared with LFD-fed mice (Figure 1G and Supplemental Figure 1A; supplemental material available online with this article; https://doi.org/10.1172/JCI180802DS1). RNAscope analysis showed increased *Has2* mRNA expression in HSCs of tumors from HFD-fed mice compared with LFD-fed mice (Figure 1H and Supplemental Figure 1, B and C). We also validated the effect of steatosis on liver metastasis from other CRC and pancreatic cancers. These models also showed increased tumor growth, ECM, HA deposition, and *Has2* expression in HFD-fed mice (Supplemental Figure 1, D and E).

We then validated the source of intratumoral CAFs using mice that expressed tdTomato under control of lecithin-retinol acyltransferase (Lrat)–Cre, which traces the HSC lineage (17–19, 22). Lrat-tdTomato–positive CAFs were increased in the tumor after HFD feeding, indicating that HSCs are the major source of intratumoral CAFs (Supplemental Figure 1F). The expression of CD44, a receptor for HA, was increased in tumors from mice fed an HFD (Supplemental Figure 1G). These findings suggest that fibrotic TME and HSC-derived CAFs in liver metastases promote tumor growth in steatotic liver, and that HSC-derived HAS2 and HA-mediated CD44 activation could play a role in developing a metastatic TME in steatotic liver.

### Loss of HSC-specific Has2 reduces steatotic liver–enhanced metastatic liver tumor growth.

**Figure 2:**
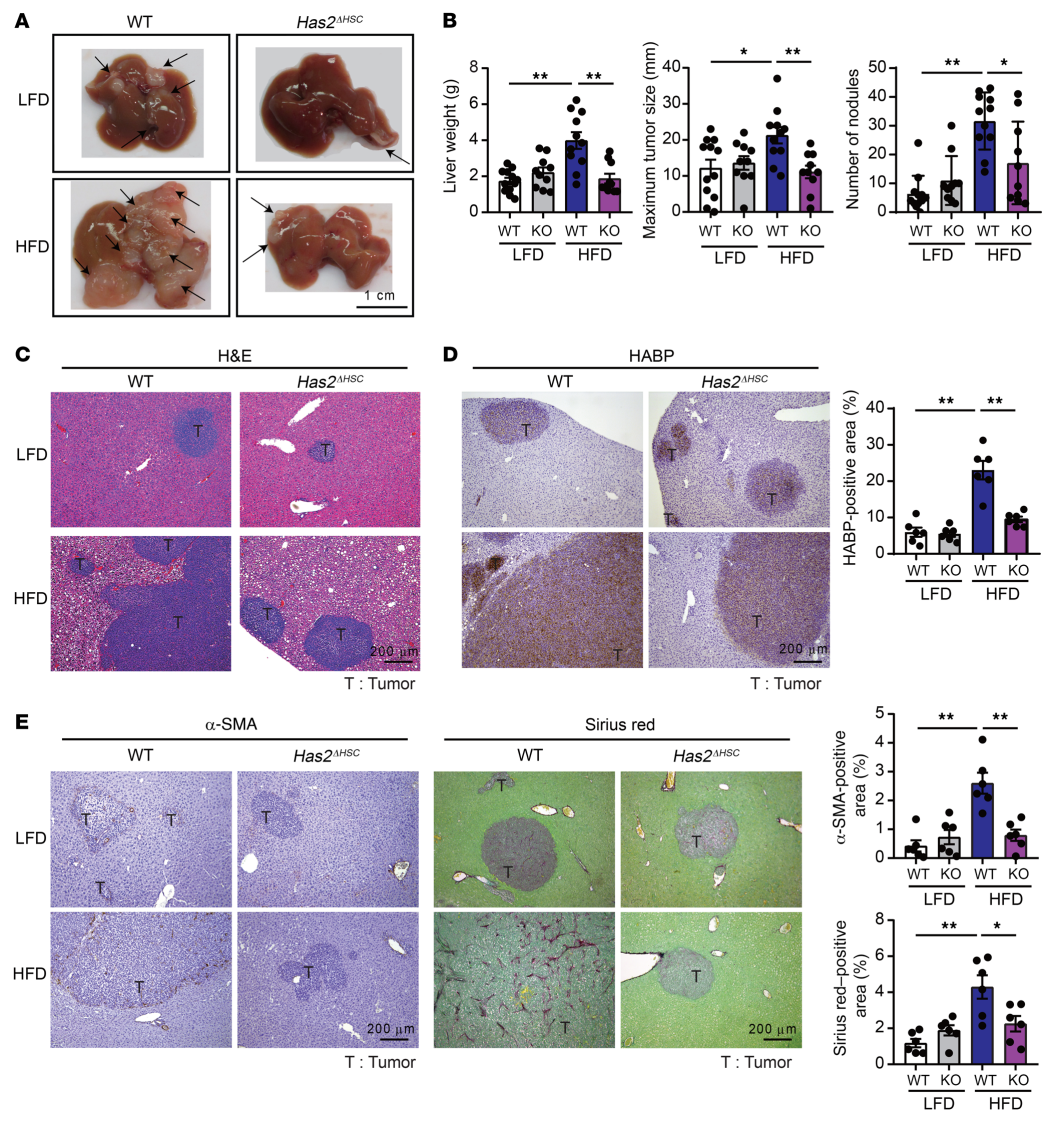
Suppression of metastatic liver tumor growth enhanced by metabolic dysfunction–associated steatotic liver disease through HSC-specific *Has2* deficiency. (**A**) Macroscopic appearance of the liver. WT and *Has2^ΔHSC^* mice were intrasplenically injected with MC38 cells after 6 weeks of either LFD or HFD feeding. Mice were maintained on their respective diets for an additional 2 weeks. Arrows indicate the tumors. Scale bar: 1 cm. (**B**) Measurement of liver weight, maximal tumor diameter, and number of nodules. (*n* = 10–12 per group.) KO, knockout. (**C**) Representative images of H&E staining of liver tissue sections. Scale bar: 200 μm. (**D**) Representative images and quantification of HABP staining. (*n* = 6.) Scale bar: 200 μm. (**E**) Representative images and quantification of α-SMA and Sirius red staining of liver tissue sections. (*n* = 6.) Scale bar: 200 μm. Data are presented as mean ± SEM. Statistical significance was calculated with 1-way ANOVA followed by Tukey’s post hoc test. *P < 0.05, **P < 0.01.

To determine how a fibrotic TME plays a role in steatotic liver–associated liver metastasis, we studied an ECM component, HA, a signaling molecule that activates cancer-promoting pathways, including CD44. Because HAS2 is the most upregulated HAS enzyme in steatotic liver–associated liver metastasis (Figure 1G and Supplemental Figure 1A) and HSCs are the cells responsible for expressing HAS2 and are the source of CAFs in the liver (20), we examined the contribution of HSC-derived HAS2 in metastatic liver tumor growth in the presence of steatotic liver using mice with HSC-specific *Has2* knockout (*Has2^ΔHSC^*). Liver metastasis was evaluated 2 weeks after MC38 CRC cell inoculation in mice fed an LFD or an HFD. The liver tumor formation was similar in WT and *Has2^ΔHSC^* mice fed an LFD; tumor growth was increased in WT mice fed an HFD compared with WT mice fed an LFD; and in mice fed an HFD, tumor growth was significantly decreased in *Has2^ΔHSC^* mice compared with WT mice (Figure 2, A–C, and Supplemental Figure 2A). The HA deposition was similar between WT and *Has2^ΔHSC^* mice fed an LFD, but the HA deposition increased in HFD-fed mice, which was reduced by HSC-specific *Has2* deletion (Figure 2D). Similarly, α-SMA expression and collagen content, representing myofibroblastic CAFs, were decreased in HFD-fed *Has2^ΔHSC^* mice compared with HFD-fed WT mice (Figure 2E), suggesting that HAS2 is crucial for developing myofibroblastic CAFs and collagen production in tumor lesions when coexistent with steatotic liver. Fat deposition developed similarly in the livers from WT and *Has2^ΔHSC^* mice fed an HFD (Supplemental Figure 2B). These results suggest that HAS2-mediated fibrotic TME plays an important role in metastatic tumor growth in the presence of steatosis. We then examined whether HA deposition contributes to the early engraftment of tumor cells to the liver by assessing liver tumors on day 3 after tumor inoculation. While we did not observe HA deposition on day 3, tumor numbers were higher in steatotic livers (Supplemental Figure 2, C and D). In addition, inhibition of HA synthesis by 4-methylumbelliferone (4-MU) did not affect the engraftment (Supplemental Figure 2, E–F). These results indicate that tumor engraftment occurred prior to HA deposition.

### LMW-HA regulates the Hippo/YAP pathway in CRC liver metastasis.

**Figure 3:**
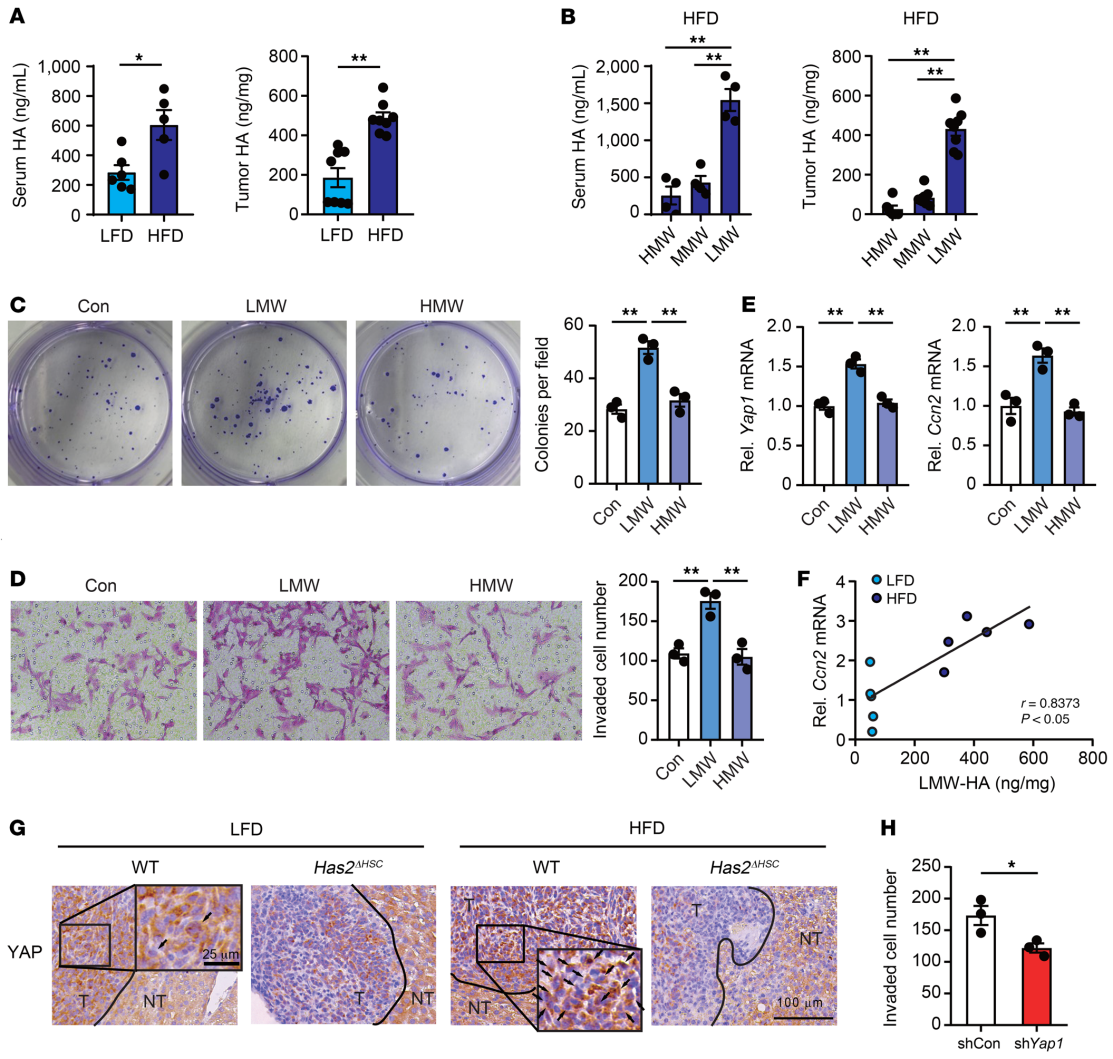
HAS2 and LMW-HA play an important role in cancer cell aggressiveness and YAP activation. (**A**) Serum HA levels (left) and tumor HA levels (right) in tumor-bearing mice on an LFD and those on an HFD. (Serum, *n* = 5–6 per group; tumor, *n* = 8 per group.) (**B**) Fractionated analysis of HA content, distinguishing between HMW-HA (>300 kDa), medium–molecular weight (MMW) HA (100–300 kDa), and LMW-HA (<100 kDa). Serum and tissue homogenate samples from HFD-fed, tumor-bearing mice were fractionated using columns. (Serum, *n* = 4; tumor, *n* = 8.) (**C** and **D**) The influence of LMW-HA and HMW-HA on the colony formation (**C**) and invasion ability (**D**) of MC38 cells. (*n* = 3.) (**E**) The effect of LMW-HA and HMW-HA on *Yap1* and *Ccn2* mRNA expression in MC38 cells. (*n* = 3.) Con, control. (**F**) The correlation between LMW-HA levels and *Ccn2* mRNA expression in tumors from mice on an LFD and on an HFD. The Pearson’s correlation coefficient (*r*) was calculated. (*n* = 5.) (**G**) Effect of HSC-specific *Has2* deletion on YAP expression in tumors from WT mice or knockout mice. (*n* = 8.) Representative images of YAP staining are shown. NT, nontumor; T, tumor. Scale bar: 100 μm. (**H**) The effect of *Yap1* knockdown in MC38 cells on LMW-HA–induced cancer cell invasion. The number of invaded cells per field is shown. (*n* = 3.) sh, short hairpin. Data are presented as mean ± SEM. Statistical significance was calculated with Student’s *t* test (**A** and **H**) and 1-way ANOVA followed by Tukey’s post hoc test (**B**–**E**). *P < 0.05, **P < 0.01.

Although our previous study demonstrated that steatotic liver–derived extracellular vesicles regulate YAP signaling through their cargo microRNAs (14), we sought to determine whether HA also plays a role in YAP activation. HA can be formed in different sizes, HMW-HA and LMW-HA, generated by hyaluronidases (23). After binding to HA receptors, such as CD44, HMW-HA and LMW-HA differentially regulate YAP signaling (21). HA concentrations in the serum and tumor were significantly higher in tumor-bearing mice fed an HFD than an LFD (Figure 3A). Under the HFD condition, LMW-HA was higher than other forms of HA in the serum and tumor of mice bearing metastatic liver tumors (Figure 3B). We determined that the expression of the HA receptors CD44 and TLR4 was significantly increased in tumors from mice fed an HFD compared with an LFD, suggesting that HA receptor signaling is augmented in liver metastasis under steatotic liver conditions (Supplemental Figure 1G and Supplemental Figure 3A). LMW-HA treatment induced proliferation and invasive capacity along with mRNA upregulation of *Yap1* and its target *Ccn2* in MC38 CRC cells, whereas HMW-HA treatment had no effect (Figure 3, C–E). *Ccn2* expression was positively correlated with LMW-HA in mouse metastatic liver tumors (Figure 3F). Correlation of YAP target genes, such as *Ccn2*, *Ccn1*, *Axl*, and *Ankrd1*, with *Has2* mRNA expression was also observed (Supplemental Figure 3B). Furthermore, nuclear translocation of YAP and mRNA expression of *Yap1* and *Ccn2* were decreased in tumors from *Has2^ΔHSC^* mice fed an HFD, suggesting a role of HAS2 and HA in the activation of YAP (Figure 3G and Supplemental Figure 3C). Subsequently, we investigated the mechanism of LMW-HA–induced YAP activation. FAK phosphorylates YAP at tyrosine 357 residues, resulting in YAP nuclear translocation (24). We examined whether FAK is involved in LMW-HA–induced YAP activation using a FAK inhibitor. FAK inhibition suppressed LMW-HA–induced nuclear translocation of YAP (Supplemental Figure 3, D and E). Lastly, we validated the role of YAP in HA-mediated cancer activity. *Yap1* silencing using short hairpin RNA (shRNA) in MC38 CRC cells reduced LMW-HA–induced CRC invasion (Figure 3H), indicating that YAP plays a role in HA-mediated CRC invasion. All these findings support the mechanism by which HAS2 and LMW-HA regulate YAP signaling to promote metastatic CRC activities under steatotic liver conditions.

### Cancer YAP signaling mediates HAS2 expression and myofibroblastic CAF development.

**Figure 4:**
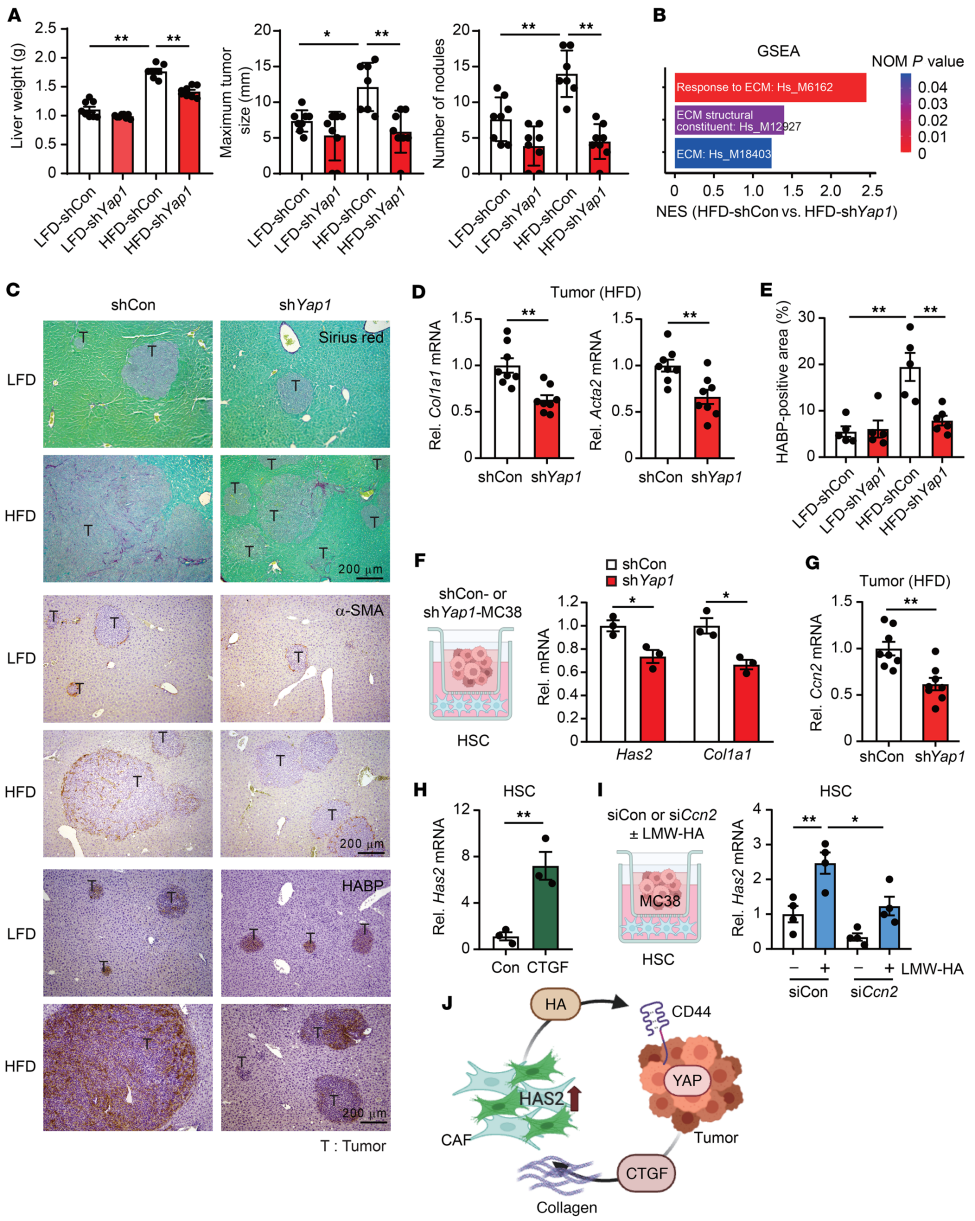
YAP knockdown attenuates CRC aggressiveness and CAF activation. (**A**) After 6 weeks of LFD or HFD feeding, MC38 cells with either shCon or sh*Yap1* were intrasplenically injected into mice. Left: Liver weight. Middle: Maximum tumor size. Right: Number of nodules. (*n* = 7–8 per group.) (**B**) Gene set enrichment analysis. NES, normalized enrichment score; NOM, nominal. (**C**) Representative Sirius red (top), α-SMA (middle), and HABP (bottom) in metastatic liver tumors. (**D**) mRNA expression of *Col1a1* and *Acta2* in tumor tissues. (*n* = 8.) (**E**) Quantification of HABP-positive area. (*n* = 5–6 per group.) (**F**) Coculture experiments. *Has2* and *Col1a1* mRNA levels in mouse primary HSCs are shown. ShCon-MC38 or sh*Yap1*-MC38 cells were placed in the upper chamber, and primary HSCs were seeded in the lower chamber. (*n* = 3.) (**G**) *Ccn2* mRNA levels in tumor tissues. (*n* = 8.) (**H**) CTGF treatment in primary HSCs. (*n* = 3.) (**I**) *Has2* mRNA levels in mouse primary HSCs. MC38 cells were transiently transfected with small interfering RNA for control (siCon) or Ccn2 (si*Ccn2*) and treated with vehicle or LMW-HA. MC38 cells were loaded in the upper chamber. HSCs were seeded in the lower chamber 1 day before coculture. Coculture lasted 48 hours. (*n* = 4.) (**J**) Illustration showing the bidirectional regulation between HSCs and CRC. Data are presented as mean ± SEM. Statistical significance was calculated with Student’s *t* test (**D** and **F**–**H**) and 1-way ANOVA followed by Tukey’s post hoc test (**A**, **E**, and **I**). *P < 0.05, **P < 0.01.

YAP is considered to be a master regulator in cancer, providing many malignant attributes (25). Our data demonstrate that YAP is a key downstream effector of HA signaling, promoting cancer activity (Figure 3). To further analyze the role of YAP in CRC liver metastasis enhanced by steatosis, *Yap1* was silenced in MC38 CRC cells and was used for the steatotic liver–associated liver metastasis model. Consistent with our previous study (14), we observed similar results that *Yap1* silencing in MC38 CRC cells reduced metastatic tumor growth enhanced by HFD feeding (Figure 4A). These newly obtained data validate our prior findings in an independent set of experiments. Bulk RNA-Seq analysis revealed that the ECM-related gene signatures were reduced when *Yap1* was silenced (Figure 4B), indicating that the cancer-derived YAP signaling not only acts as a downstream effector of HA but also promotes CAF activity to regulate a fibrotic TME. Collagen deposition and myofibroblastic CAF infiltration were reduced by knockdown of *Yap1* in tumors of steatotic liver as assessed by Sirius red and α-SMA staining, respectively (Figure 4C). The mRNA expression of *Col1a1* and *Acta2* was also reduced in *Yap1*-silenced tumors from HFD-fed mice compared with that in control tumors from HFD-fed mice (Figure 4D). The HA deposition increased by an HFD was significantly reduced by knockdown of *Yap1* (Figure 4, C and E). To prove the critical role of YAP activity in MC38 CRC cells for CAF activity, HSCs were cocultured with control and *Yap1*-silenced MC38 cells. *Yap1* silencing in MC38 CRC cells significantly reduced the expression of *Has2* and *Col1a1* in HSCs compared with HSCs cocultured with control MC38 cells (Figure 4F). These data suggest that YAP-mediated secreted factor(s) contribute to CAF activation. Because connective tissue growth factor (CTGF), encoded by *Ccn2*, and cysteine-rich angiogenic inducer 61 (CYR61) are secreted factors downstream of YAP signaling (Figure 4G), we investigated whether CTGF or CYR61 is responsible for CAF activation in liver metastasis. HSCs were treated with either CTGF or CYR61. Both CTGF and CYR61 increased *Col1a1* and *Timp1* mRNA expression (Supplemental Figure 4). However, only CTGF-treated HSCs showed increased *Has2* mRNA expression (Figure 4H and Supplemental Figure 4). To evaluate the effect of HA-mediated CTGF production from MC38 CRC cells on *Has2* expression in HSCs, HSCs were cocultured with control and *Ccn2*-silenced MC38 cells with or without LMW-HA treatment (Figure 4I). When MC38 cells were stimulated with LMW-HA, *Has2* mRNA expression levels were increased in HSCs. *Ccn2* knockdown in MC38 cells abolished this effect. Taken together, these results indicate that YAP signaling in cancer cells, enhanced by steatotic liver through HAS2-mediated HA production, feeds back to promote CAF activation, including HAS2 upregulation, to further increase the fibrotic TME (Figure 4J).

### Increase in CAF activity by steatotic liver contributes to a prometastatic immune TME.

**Figure 5:**
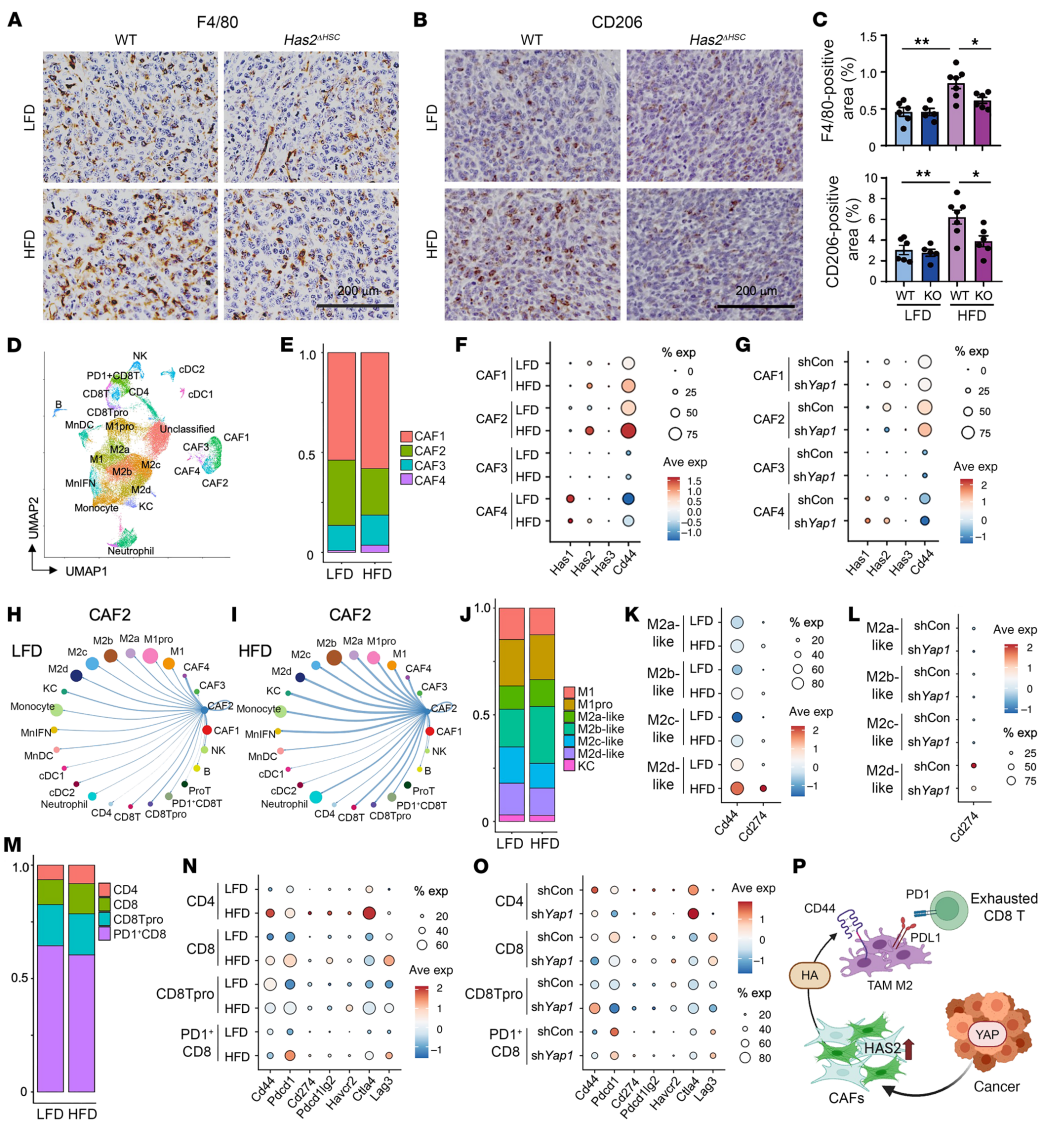
CAF-derived HAS2 and cancer-derived YAP contribute to a prometastatic immune TME in steatotic liver. (**A** and **B**) Representative immunohistochemistry images for F4/80 (**A**) and CD206 (**B**) from tumors in Figure 2. Scale bars: 200 μm. (**C**) Quantification of F4/80-positive (top) and CD206-positive (bottom) areas. (*n* = 5–7 per group.) Data are presented as mean ± SEM. Statistical significance was calculated with 1-way ANOVA followed by Tukey’s post hoc test. *P < 0.05, **P < 0.01. (**D**) Tumor-infiltrating CAF and immune cell populations. Uniform manifold approximation and projection (UMAP) of single-cell RNA-Seq from 46,577 cells showing 25 clusters determined by integrated analysis, colored by cluster. Cells were from metastatic liver tumors of LFD-fed and HFD-fed mice. (*n* = 3 per group.) (**E**, **J**, and **M**) The proportion of CAF (**E**), M1 and M2 (**J**), and T cell (**M**) clusters in metastatic liver tumors of LFD-fed and HFD-fed mice. (**F** and **G**) Expression of *Has1*, *Has2*, *Has3*, and *Cd44* genes (columns) by specific CAF subpopulations (rows). Dot size represents the cell fraction within the CAF subpopulations. Fill color indicates average expression (Ave. exp.). (**H** and **I**) CellChat (53) receptor-ligand analysis of the predicted intercellular communication networks for cells from metastatic liver tumors of LFD-fed and HFD-fed mice. Arrows are proportional to the interaction strength between CAF2 and other cell clusters; node size indicates the number of cells within that population. (**K** and **L**) Expression of *Cd44* and *Cd274* genes (columns) by specific M2 subpopulations (rows). Dot size represents the cell fraction within the M2 subpopulations. (**N** and **O**) Expression of key immunomodulatory genes (columns) by specific T cell subpopulations (rows). (**P**) Proposed model representing cancer YAP regulation of HSC-derived HAS2 for the immunosuppressive TME in steatotic liver.

TAM M2 polarization is associated with a prometastatic immunosuppressive microenvironment in steatotic liver (13, 14). As an increase in HAS2 expression by steatotic liver promotes metastatic liver tumor growth, we investigated the impact of HAS2 on M2-TAM infiltration using *Has2^ΔHSC^* mice. The increase in infiltration of F4/80-positive and CD206-expressing M2-polarized TAMs by steatotic liver was decreased in mice with *Has2* deletion in HSCs (Figure 5, A–C). These results indicate that CAF-derived HAS2 contributes to the infiltration of M2-polarized TAMs to metastatic lesions in steatotic liver. We further analyzed CAF and immune cell populations in the TME using single-cell RNA-Seq. Unsupervised clustering identified 25 clusters, including 4 CAF, 2 M1, 4 M2, and 4 T cell clusters (Figure 5D and Supplemental Figure 5, A–C). Although an HFD slightly decreased the proportion of CAF2, the total CAF number was doubled (Figure 5E and Figure 1F), and the CAF2 cluster increased the expression of HAS2 and CD44 with an HFD (Figure 5F). These data underscore that the number of total HAS2-expressing CAF2 cells is increased in the steatotic liver. Furthermore, as is corroborated by the in vitro study showing that cancer-derived YAP regulates HAS2 in HSCs (Figure 4F), silencing *Yap1* in cancer cells reduced *Has2* expression in the CAF2 cluster (Figure 5G). We then performed cell-cell interaction analysis between HAS2-expressing CAF2 and other immune cell clusters (Figure 5, H and I). CAF2 and macrophage/monocyte clusters showed stronger interactions for an HFD than those for an LFD (Figure 5, H and I). Among macrophage subsets, the proportion of M2b-like cells was increased (Figure 5J). Because M2d-like cells expressed high levels of *Cd44* (Figure 5K), the ligand-receptor interaction between CAF2 and M2d-like cells could be mediated through HAS2/HA/CD44. Additionally, M2d cells highly expressed *Cd274* (which encodes programmed death–ligand 1 [PD-L1]), but *Cd274* expression was reduced when *Yap1* was silenced in cancer cells (Figure 5L). Although CAF3 expressed *Cd274*, the interactions between CAF3 and T cells were modest (Supplemental Figure 5, D–F). In contrast, increased interactions between M2d and T cells were observed in steatotic liver (Supplemental Figure 5, G and H); this could be due partly to the increased expression of *Cd274* in M2d cells and *Pdcd1* (which encodes programmed cell death protein 1 [PD-1]) in T cells (Figure 5, K, M, and N). *Cd274* expression in M2d cells and *Pdcd1* expression in T cells were decreased when cancer *Yap1* was silenced (Figure 5, L and O). In summary, these findings suggest that cancer-derived YAP activity contributes to the HAS2/HA/CD44 interaction between CAFs and TAMs and the enhanced interaction of PD-L1 and PD-1 between TAMs and T cells (Figure 5P). These CAF–TAM–T cell interactions promote a prometastatic immunosuppressive TME in steatotic liver.

### Increased myofibroblastic CAF infiltration is associated with immunosuppressive TAM and T cells in patients with CRC liver metastasis with steatotic liver.

**Figure 6:**
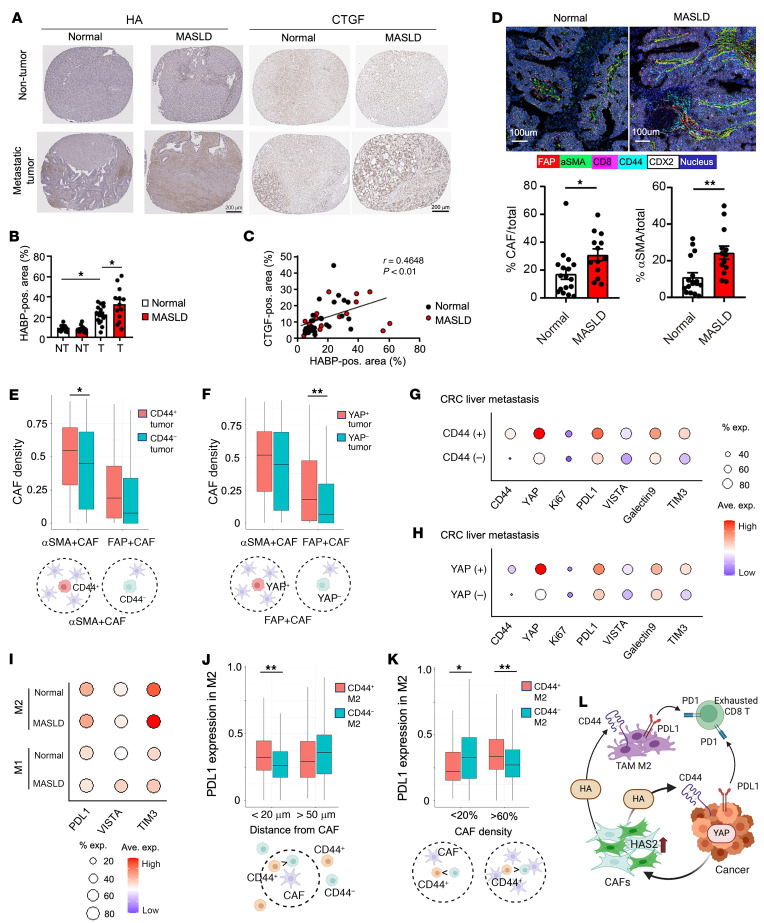
Increased CAF infiltration and immunosuppressive TAM and T cell phenotypes in patients with CRC liver metastasis with steatotic liver. (**A**) Representative images for HABP staining (left) and CTGF staining (right) using tissue microarray sections of metastatic CRC patients. Scale bars: 200 μm. (**B**) Quantification of HABP-positive area. (Normal, *n* = 16; MASLD, *n* = 13.) NT, nontumor; T, tumor. (**C**) Correlation between HABP-positive area and CTGF-positive area. Pearson’s correlation coefficient (*r*) was calculated. (Normal, NT, *n* = 15; MASLD, NT, *n* = 12; normal, tumor, *n* = 14; MASLD, tumor, *n* = 11.) (**D**) Top: Representative IMC images for metastatic liver tumors for FAP, α-SMA, CD8, CD44, and CDX2 expression. Scale bars: 100 μm. Bottom: Per-patient proportions of CAFs and α-SMA–positive CAFs. (Normal, *n* = 17; MASLD, *n* = 13.) (**E** and **F**) Spatial analysis of IMC data to evaluate density of CAFs surrounding CD44-positive or -negative (**E**) or YAP-positive or -negative (**F**) cancer cells. (**G** and **H**) Dot plot for expression of CD44, YAP, Ki67, and immunomodulatory molecules (columns) on CD44-positive and -negative (**G**) or YAP-positive and -negative (**H**) cancer cells (rows). Dot size represents the cell fraction within each cell population. Fill color indicates average expression (Ave. exp.). (**I**) Dot plot for expression of immunomodulatory molecules (columns) by macrophage subpopulations from patients with or without MASLD (rows). (**J** and **K**) Spatial analysis of IMC data to evaluate the relationship between macrophage PD-L1 expression and macrophages’ distance from CAFs (**J**) or density of CAFs (**K**). (**L**) Illustration of the proposed model. Data are shown as mean ± SEM (**B** and **D**) or mean ± SD (**E**, **F**, **J**, and **K**). Statistical significance was calculated with 1-way ANOVA followed by Tukey’s post hoc test (**B**) or with 2-tailed Student’s *t* test (**D**) or generalized linear models using the sample as a clustering variable to obtain robust standard error (**E**, **F**, **J**, and **K**). *P < 0.05, **P < 0.01.

To translate our findings in mice to humans, we used tissue microarrays containing tissue specimens from 30 patients with CRC liver metastasis with and without steatotic liver (Supplemental Figure 6A) (14). First, we examined HA deposition. While almost no HA deposition was observed in nontumor liver tissues, tumors from patients with normal livers had high levels of HA deposition; the level of HA deposition in tumors was further increased in patients with steatotic liver (Figure 6, A and B). As we determined CTGF as an HA inducer (Figure 4), we assessed CTGF expression. CTGF levels were increased in tumors from patients with steatosis, and there was a significant correlation between HA and CTGF expression (Figure 6, A and C), corroborating our preclinical data. Then, we conducted imaging mass cytometry (IMC) using 42 metal-conjugated antibodies (14). We performed segmentation and phenotyping to identify liver-constituting and TME-constituting cell populations at the single-cell level, including hepatocytes, immune cells, CAFs, endothelial cells, and cancer cells (Figure 6D and Supplemental Figure 6, B–D) (14). The numbers of total CAFs and myofibroblastic α-SMA–positive CAFs were increased in patients with metabolic dysfunction–associated steatotic liver disease (MASLD) (Figure 6D). Both CD44-positive and YAP-positive tumors had higher densities of α-SMA–positive and fibroblast activation protein–positive (FAP-positive) CAFs than CD44-negative and YAP-negative tumors, respectively (Figure 6, E and F). The data suggest that the α-SMA–positive CAF effect on CD44-positive tumors is mediated through HA, while YAP signaling acts on FAP-positive CAF recruitment. Also, CD44 and YAP expression was associated with the expression of PD-L1 and other immune checkpoint molecules in metastatic CRC cells (Figure 6, G and H), whereas PD-L1 expression was not associated with the distance and the density of CAFs near tumors (Supplemental Figure 6C). CAFs, TAMs, and T cells had higher CD44 expression than other cells (Supplemental Figure 6D). The expression of PD-L1, VISTA, and TIM3 was unchanged in CAFs between normal and MASLD livers (Supplemental Figure 6E). In contrast, the expression of PD-L1, VISTA, and TIM3 was higher in M2-TAMs and CD8^+^ T cells of MASLD specimens, and CD8^+^ T cells also had higher expression of PD-1 in MASLD specimens (Figure 6I and Supplemental Figure 6F). Notably, PD-L1 expression in M2-TAMs was positively associated with CD44 expression when M2-TAMs were close to CAFs as well as when the CAF density was high, suggesting the contribution of CAF-derived HA/CD44 signaling to PD-L1 expression in M2-TAMs (Figure 6, J and K). Taken together, these findings suggest that, in MASLD, CAF-derived HA acts on CD44-expressing TAMs and CRC cells to regulate PD-L1 expression; PD-L1 expression on M2-TAMs is associated with the distance and the density of CAFs near M2-TAMs; and the increased PD-L1 binds to PD-1 expressed on CD8^+^ T cells to support an immunosuppressive TME in metastatic foci in the presence of steatosis (Figure 6L).

### Administration of 4-MU or 4-MUG inhibits the growth of metastatic cancer enhanced by steatotic liver.

**Figure 7:**
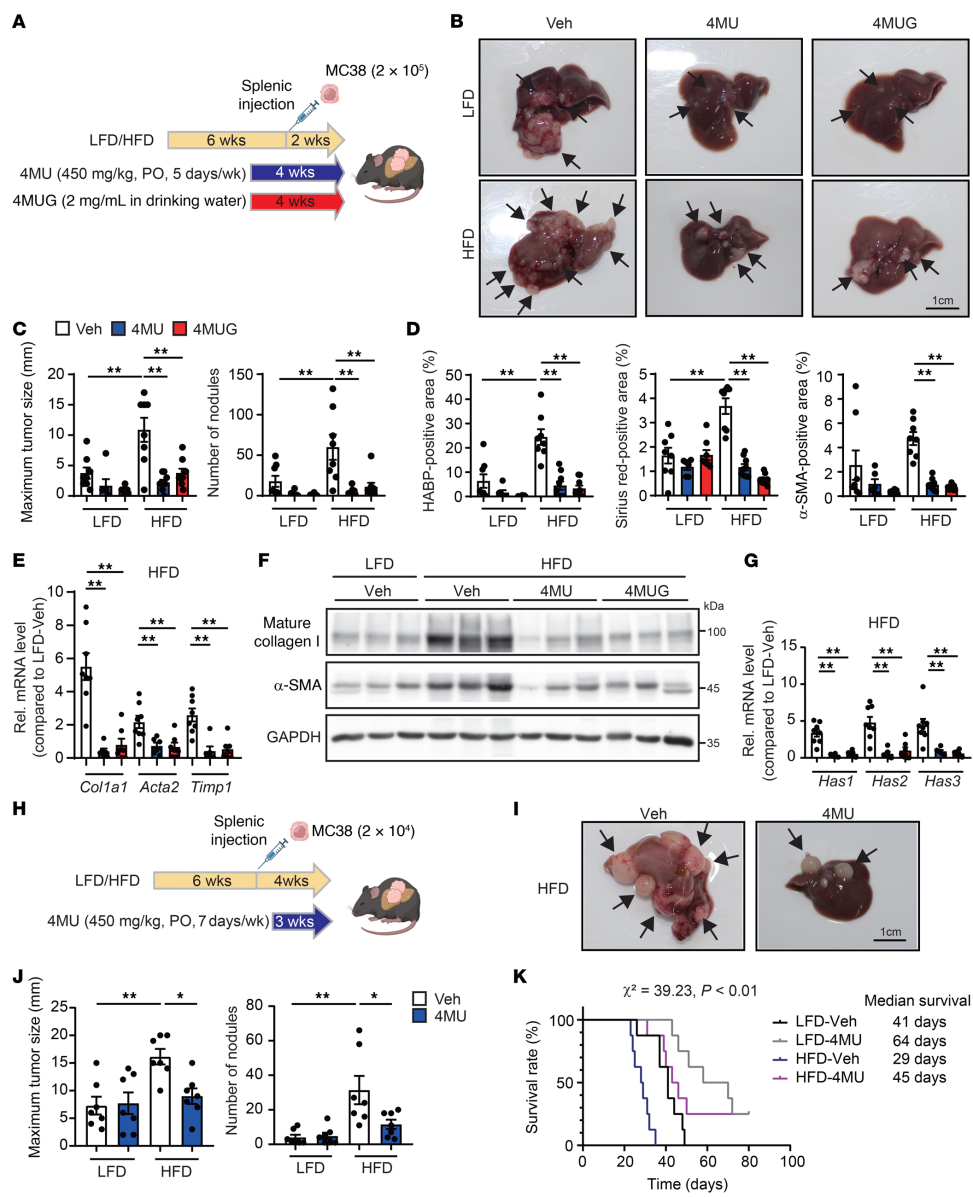
Inhibition of HA synthesis alleviates metastatic liver tumor growth and CAF activation in the steatotic liver disease condition. (**A**) In vivo preventive experimental protocol. 4-Methylumbelliferone (4-MU) was administered orally (PO) at 450 mg/kg, 5 times a week for 4 weeks, while 4-methylumbelliferyl glucuronide (4-MUG) was provided in drinking water at 2 mg/mL for 4 weeks. (**B**) Macroscopic appearance of the liver. Arrows indicate tumor sites. Veh, vehicle. Scale bar: 1 cm. (**C**) Analysis of maximal tumor diameter and number of nodules. (*n* = 6–9 per group.) (**D**) Quantitative assessment of HABP-, Sirius red–, and α-SMA–positive areas. (*n* = 6–9 per group.) (**E**) Measurement of mRNA expression levels for profibrogenic genes in HFD-fed mice treated with the respective drugs. (*n* = 6–8 per group.) (**F**) Western blot analysis of mature collagen I and α-SMA. (**G**) Evaluation of mRNA expression levels for *Has1*, *Has2*, and *Has3*. (*n* = 6–8 per group.) (**H**) In vivo treatment experimental protocol. (**I**) Macroscopic appearance of the liver from tumor-bearing mice. Scale bar: 1 cm. (**J**) Quantification of the maximal tumor diameter and the number of tumor nodules. (*n* = 7 per group.) (**K**) Kaplan-Meier survival curves. Statistical significance was determined using the log-rank test. (*n* = 8 per group.) Data are presented as mean ± SEM. Statistical significance was calculated with 1-way ANOVA followed by Tukey’s post hoc test (**C**–**E**, **G**, and **J**). *P < 0.05, **P < 0.01.

**Figure 8:**
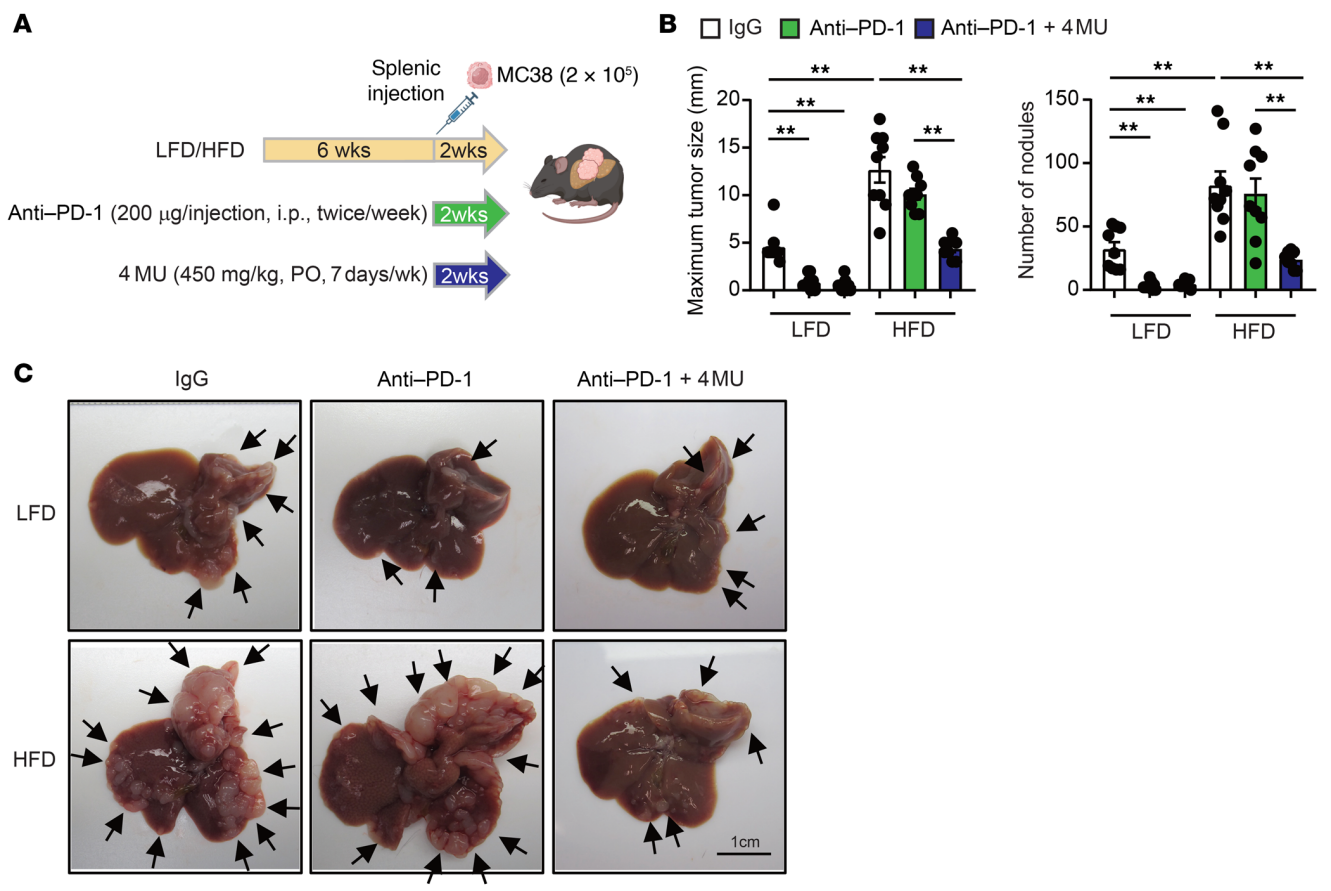
Improved effects of anti–PD-1 antibody treatment in combination with HA synthesis inhibition on metastatic liver tumor growth in the steatotic liver disease condition. (**A**) Experimental protocol for the in vivo combination treatment of 4-MU and anti–PD-1 antibody. 4-MU was administered orally (PO) at 450 mg/kg daily for 2 weeks, while anti–PD-1 antibody (200 μg) was administered intraperitoneally (i.p.) every 3 days for a total of 4 injections. (**B**) Analysis of maximal tumor diameter and number of nodules. (*n* = 8–9 per group.) Data are presented as mean ± SEM. Statistical significance was calculated with 1-way ANOVA followed by Tukey’s post hoc test. **P < 0.01. (**C**) Macroscopic appearance of the liver. Arrows indicate tumor sites. Scale bar: 1 cm. IgG, control IgG.

Last, we tested the potential of pharmacological inhibition of HA synthesis by 4-methylumbelliferone (4-MU) and 4-methylumbelliferyl glucuronide (4-MUG) to inhibit the growth of liver metastasis under the steatotic liver condition. C57BL/6 male WT mice were fed an LFD or an HFD diet and received a splenic injection of MC38 cells at the 6-week mark. We tested preventive and treatment interventions. For the prevention study, 1 group of mice received 450 mg/kg 4-MU orally, and another group of mice received 2 mg/mL 4-MUG in their drinking water (Figure 7A). In LFD-fed mice, 4-MU and 4-MUG tended to reduce metastatic tumor growth, but this was not statistically significant (Figure 7, B and C). In HFD-fed mice, 4-MU and 4-MUG treatment significantly inhibited tumor growth, HA deposition (by HA-binding protein [HABP] staining), collagen content (by Sirius red staining, mRNAs, and protein), and myofibroblastic CAFs (by α-SMA immunostaining, mRNA, and protein) (Figure 7, B–F, and Supplemental Figure 7, A–C). The 4-MU and 4-MUG treatment suppressed *Has1*, *Has2*, and *Has3* mRNA expression in HFD conditions (Figure 7G). We then examined YAP signaling. Phosphorylated YAP (pSer^127^-YAP) has reduced transcriptional activity (26). We observed a significant reduction in pSer^127^-YAP in tumors of HFD-fed mice compared with LFD-fed mice, along with a reciprocal increase in total YAP (Supplemental Figure 7D). When mice with HFD were treated with 4-MU or 4-MUG, there was an increase in pSer^127^-YAP in the tumor, while total YAP decreased (Supplemental Figure 7D). Also, 4-MU or 4-MUG treatment downregulated YAP target genes, such as *Ccn1*, *Axl*, *Ankrd1*, and *Ccl2*, in steatotic liver tumors, mirroring the effects on *Yap1* mRNA expression (Supplemental Figure 7E). The increased presence of F4/80-positive TAMs in the tumors of HFD-fed mice was diminished by the inhibition of HA synthesis of 4-MU or 4-MUG (Supplemental Figure 7C). 4-MU or 4-MUG attenuated the expression of M2 macrophage–related markers and partially attenuated the expression of M1 macrophage markers (Supplemental Figure 7F). These results collectively indicate that 4-MU and 4-MUG treatment reduced the activity of YAP and its target genes, which contribute to suppressing metastatic tumor growth in MASLD. Additionally, we examined the efficacy of the treatment regimen by starting the oral administration of 4-MU one week after tumor inoculation and continuing it for an additional 3 weeks (Figure 7H). In the treatment regimen, we still observed the tumor suppression effect of 4-MU in the steatotic liver condition (Figure 7, I and J, and Supplemental Figure 7G). 4-MU treatment reduced *Has2* mRNA, profibrogenic genes, and YAP target genes in the tumors of HFD-fed mice (Supplemental Figure 7H). HFD-fed mice exhibited reduced survival compared with LFD-fed mice, with a median survival of 29 days versus 41 days. Notably, 4-MU treatment extended survival in both LFD- and HFD-fed groups (Figure 7K). Lastly, we tested potential combination therapy of 4-MU and anti–PD-1 antibody. Anti–PD-1 therapy was effective in non-steatotic liver conditions, but it was ineffective in the steatotic liver (Figure 8). However, in combination with 4-MU, anti–PD-1 therapy showed a dramatic effect in suppressing metastatic liver tumor growth (Figure 8), suggesting the potential of intervention with this combination.

## Discussion

MASLD is the leading cause of chronic liver disease and affects 30% of the population worldwide (27). The prognosis of MASLD is determined by its progression to metabolic dysfunction–associated steatohepatitis, fibrosis, cirrhosis, and the development of hepatocellular carcinoma (8, 28). MASLD also increases the incidence of cardiovascular events (29) and cancers in extrahepatic organs, such as stomach, colon, pancreas, prostate, and breast (7, 8). Liver is the most common metastasis site for CRC, and liver metastases substantially worsen CRC prognosis (1, 2, 5). We previously reported that steatotic hepatocyte–derived extracellular vesicles play pivotal roles in CRC liver metastasis by creating an immunosuppressive TME via YAP and CYR61 (14). CYR61 is a regulator of M2 macrophage infiltration and polarization, which interacts with PD-1–positive CD8^+^ T cells to create immunosuppressive TME. In the present study, we further explored a new mechanism of how MASLD promotes CRC liver metastasis. Here, we found that less desmoplastic CRC liver metastases become more desmoplastic when the liver is steatotic. Consistent with our previous report showing that HSCs are the cells responsible for producing HA through the upregulation of HAS2 (20), HSC-derived CAFs actively produce HA through HAS2 upregulation in the steatotic liver. CAF-derived HA contributes to metastatic tumor growth mediated by YAP, likely through the CD44/FAK axis. Cancer-derived YAP conversely activates CAFs through the production of CTGF, a YAP downstream target. This bidirectional regulation between CAFs and cancer cells further promotes metastatic tumor growth. Our single-cell RNA-Seq and IMC analyses further revealed that CAFs mediate PD-L1 upregulation in the nearest M2-TAMs, likely through the HA/CD44 interaction. Also, CD44 positivity and YAP positivity affect PD-L1 expression and CAF density in metastatic CRC cells. The upregulated PD-L1 in M2-TAMs and cancer cells further induces T cell exhaustion via PD-1. Last, we suggest that pharmacological HAS inhibition by 4-MU or 4-MUG and combination therapy with anti–PD-1 antibody could be a treatment option for patients with CRC liver metastasis with MASLD. In Supplemental Figure 8, we summarize the underlying mechanisms reported previously and proposed here.

Desmoplasia, which involves the deposition of fibrous ECM around a tumor, is observed frequently in pancreatic cancer and its liver metastases (30). The fibrous ECM deposition in CRC liver metastasis is variable (31). Increased fibrous ECM deposition is associated with poor prognosis and resistance to chemotherapies (30). Our data showed that steatotic liver enhanced ECM deposition and CAF myofibroblastic differentiation, which is further associated with the aggressiveness of metastatic tumor growth in the liver. FGF-2 and PDGF have been implicated in promoting CRC metastasis. Elevated FGF-2 levels have been shown to enhance tumor cell proliferation, angiogenesis, and migration, contributing to increased metastatic potential (32). Similarly, PDGF signaling in CRC and PDGF receptor signaling in CAFs promote CRC metastasis (33, 34). Our data showed that the expression of FGF-2 and PDGF, growth factors known to activate HSCs (35), was elevated in the tumors under steatotic liver conditions (Figure 1E), suggesting their contribution to the fibrotic TME and metastatic niche formation. The contribution of the major ECM component collagen to tumor growth is complex. Intact collagens inhibit, whereas matrix metalloproteinase–degraded collagen promotes, pancreatic cancer growth (30). Similarly, HA comprises different sizes that have opposing biological activities (20, 21). HAS2 controls HA synthesis in HSCs, and HAS2-derived HA promotes fibrosis progression and cancer aggressiveness in breast, esophageal, bile duct, pancreatic, and other cancers (17, 18, 20, 36–38). Our data demonstrated that HAS2 expressed in HSC-derived CAFs contributes to HA production and promotes the growth of CRC liver metastasis under the steatotic liver condition. Other HAS isoenzymes, such as HAS1 or HAS3, could also contribute to HA deposition since HA still remained detectable in HAS2-null mice. HAS1 and HAS3 expression is indeed increased in metastatic tumors of HFD-fed mice compared with LFD-fed mice. However, HAS2 shows the most pronounced upregulation among the 3 enzymes. It is likely that HAS1 and HAS3 act cooperatively with HAS2 to support HA deposition and tumor growth. Nonetheless, our data suggested that HAS2 plays the dominant role in MASLD-associated liver metastasis. HAS2 primarily synthesizes HA as HMW forms, and HA is then degraded into LMW-HA by hyaluronidases, including hyaluronidases 1–4 and cell surface hyaluronidase (CEMIP2, also known as TMEM2). HMW-HA and LMW-HA have different biological effects (20, 21, 23). HMW-HA has antiinflammatory and antiproliferative properties, whereas LMW-HA promotes cell migration, proliferation, and immune cell influx (39). Our study revealed that LMW-HA is procancerous, and LMW-HA is the dominant form in the serum and tumor of steatotic livers. The precise mechanism that converts HA from HMW to LMW forms in CAFs and cancers remains unknown. TMEM2 is a candidate for this process, as its levels were found to be elevated in metastatic tumors of HFD-fed mice (Figure 1E); however, further investigation is required.

In addition to MASLD, other liver diseases, including alcohol-associated liver disease (ALD), hepatitis B, and cirrhosis in humans and ALD and carbon tetrachloride–induced liver injury in mice (40–43), also enhance liver metastasis growth. In our previous and present studies, we determined that extracellular vesicle production and ECM deposition are mechanisms of enhanced liver metastasis in MASLD. Additional factors, including lipid metabolism, inflammatory signaling, and immune cells, are also involved in this process (44, 45). Further research is needed to determine whether other mechanisms are involved in promoting liver metastasis in MASLD and other liver disease.

The primary receptors for HA are CD44 and TLR 4 (20, 23). CD44 is a cancer stem cell marker and has a cell-adhesive capacity for interaction with cancer cells. Our data demonstrated that CD44 expression was elevated in cancer cells under steatotic liver conditions and was closely associated with the nearest CAF density that may amplify the HA-mediated signaling pathway to enhance the proliferation, invasion, and metastasis of CRC cells in the liver. CD44 is also expressed in myeloid and lymphoid cells. Our single-cell analysis revealed that steatotic liver promoted CD44 and PD-L1 expression in M2-TAM and cancer cells, which was associated with the nearest CAF distance and density. This suggests that CAF-derived HA interacts with the nearest M2-TAMs and cancer cells through CD44, promoting the upregulation of PD-L1 together with PD-1–expressing T cells to create a protumorigenic immunosuppressive TME in steatotic liver.

Hippo/YAP signaling is a pivotal protumorigenic and prometastatic factor in many cancers through interaction with the transcriptional enhanced associate domain (TEAD) transcription factors (25, 46–48). YAP regulates tumor growth and progression both intrinsically and extrinsically. YAP signaling promotes cancer cell growth and survival, which is in line with our findings that YAP silencing in cancer cells diminishes tumor growth in non-steatotic livers (Figure 4A). Simultaneously, YAP senses changes in the external environment and responds to the signals. In MASLD, CAF-derived HAS2 and LMW-HA augmented YAP nuclear translocation and YAP downstream gene expression. FAK is responsible for LMW-HA–induced YAP nuclear translocation. YAP not only acts downstream of the HA/CD44 signaling pathway but also plays a role in the activation of CAFs by producing CTGF (Figure 5P). Indeed, liver metastases by YAP-silencing CRC cells had reduced CAF activity, collagen and HA deposition, and CTGF expression. In HSCs, CTGF upregulates HAS2 expression. Additionally, our IMC data suggested that CD44 and YAP reciprocally regulate in cancer cells and that CD44 expression and YAP expression are associated with PD-L1 expression and CAF density in cancer regions. These data collectively suggest bidirectional regulation between CAFs and cancer cells through HAS2/HA/CD44/YAP signaling. Further study could identify more precise molecular mechanisms of the bidirectional regulation between CAFs and cancer cells in CRC liver metastasis.

Because our study revealed the procarcinogenic and prometastatic role of HAS2 and HA, modulation of HAS2 and HA production can be a therapeutic strategy to inhibit metastatic tumor growth in steatotic liver. Also, ECM deposition prevents the delivery of anticancer drugs to cancer lesions. Therefore, reduction of ECM deposition may enhance the efficacy of other anticancer drugs. However, clinical trials for pancreatic cancer using a hyaluronidase, PEGPH20, to promote the reduction of HA deposition did not show a favorable result (49). This could be explained by the fact that PEGPH20 degrades HA to generate protumorigenic LMW-HA. In contrast, the approach that we tested in this study was to inhibit HA synthesis with the HAS inhibitors 4-MU and 4-MUG. We previously reported that 4-MU inhibits HA synthesis and liver fibrosis progression (20, 50). Our present study showed that 4-MU or 4-MUG treatment inhibited the expression of all three HAS enzymes, HA synthesis, CAF activity, and M2 macrophage infiltration (Figure 7). Hepatic macrophages and TAMs polarize into M2 macrophages in metastatic tumors (13, 14). An increase in M2 macrophages is associated with poor prognosis of patients with CRC liver metastasis (51). We also demonstrated the importance of M2-TAM polarization in metastatic liver tumor growth in MASLD (13, 14). Together with our data in the present study, these findings underscore that inhibition of HA synthesis suppresses tumor growth in steatotic liver by suppressing HA deposition, CAF activation, and M2-TAM infiltration. We expect that, unlike PEGPH20, our approach would not lead to the detrimental effect. In addition, although anti–PD-1 therapy is ineffective in steatotic livers, we found that combination therapy of 4-MU and anti–PD-1 antibody effectively suppressed metastatic tumor growth even in steatotic liver conditions. The combination therapy can be a potential therapy for patients with MASLD and liver metastasis.

In summary, steatotic liver is associated with an enhanced fibrotic TME, comprising myofibroblastic CAFs and HA deposition, that contributes to metastatic tumor growth. Additionally, the cancer cell activity enhanced by CAFs and HA further creates a feedforward loop to promote CAF activity, ECM production, and an immunosuppressive TME under steatotic liver conditions. The underlying molecular mechanisms of metastatic tumor growth may differ in normal and steatotic liver conditions. As the number of patients with cancer with basal steatotic liver is increasing, understanding the precise molecular mechanism of metastatic tumor growth in both normal and MASLD liver could help provide more appropriate clinical management for those patients. Although underlying chronic liver disease may inhibit the efficacy of anticancer therapies, including anti–PD-1 therapy, 4-MU and 4-MUG have the potential to suppress steatotic liver–associated metastatic tumor growth. The pharmacological inhibition of HA synthesis can be considered as an option to treat patients with metastatic cancer with steatotic liver.

## Methods

Further information can be found in Supplemental Methods.

### Sex as a biological variable.

Our study examined only male mice because male animals exhibited less variability in the phenotype of hepatic steatosis. Sex was not considered as a biological variable. While our study focused on male mice, the findings may be relevant to both sexes.

### Cell line.

MC38 mouse CRC cells were a gift from Michael Karin (UCLA San Diego, La Jolla, California, USA) (14). CMT93 mouse CRC cells were obtained from the American Type Culture Collection. Pan02 mouse pancreatic cancer cells were a gift from Robert Schwabe (Columbia University, New York, New York, USA) (18). Cells were cultivated in normal Dulbecco’s modified Eagle medium without pyruvate (MC38 and CMT93) or RPMI 1640 (Pan02), supplemented with 10% fetal bovine serum (FBS), and 1% penicillin/streptomycin at 37°C in a humidified 5% CO_2_ incubator. Before use, FBS underwent heat inactivation. Mycoplasma contamination was checked, and cell line authenticity was confirmed through authentication by the American Type Culture Collection. Previously, stably transfected MC38 cells expressing either shCon (control) or sh*Yap1* (*Yap1* knockdown) were established via puromycin selection (14).

### Mice.

Seven-week-old C57BL/6 male mice were purchased from The Jackson Laboratory or KOATECH Inc. *Lrat*-Cre Tg mice expressing tdTomato were provided by Robert Schwabe (Columbia University). Our study examined male mice because male animals exhibited less variability in the phenotype of hepatic steatosis. To generate mice with HSC-specific *Has2* knockout, *Lrat*-Cre Tg and *Has2^fl/fl^* mice were crossed, as previously described (20, 22). Genetically modified mice underwent at least 10 generations of backcrossing onto the C57BL/6 background. Upon reaching 8 weeks of age, mice were randomly assigned to either a low-fat diet (LFD; PicoLab Rodent Diet 20, catalog 5053) or a high-fat diet containing 60% of calories from fat (HFD; Research Diets, catalog D12492, or Bio-Serv, catalog S3282). The mice underwent an 8-week dietary intervention period. Food intake and body weight were measured weekly. Mice were housed in the same specific pathogen–free conditions within the animal facility at Cedars-Sinai Medical Center or at Kangwon National University Animal Laboratory Center. All animal studies adhered to the National Institutes of Health recommendations as delineated in the *Guide for the Care and Use of Laboratory Animals* (National Academies Press, 2011).

### Human specimens.

Biospecimens were obtained from patients diagnosed with CRC liver metastasis and coexisting MASLD, as well as from CRC patients without MASLD (‘normal’ livers), who underwent surgical resection or tissue biopsy at Cedars-Sinai Medical Center (14). Comprehensive clinical, demographic, and pathological data were retrospectively extracted from electronic medical records for subsequent analysis (Supplemental Table 1) (14). A tissue microarray block was created using primary CRC, CRC liver metastasis, and adjacent non-cancer liver tissues from 17 patients without MASLD and 13 patients with MASLD as previously described, and applied for IMC and staining (14).

### In vivo model of liver metastasis by splenic injection.

A murine model of liver metastasis was established by splenic injection of MC38, CMT93, and Pan02 cells, as previously described (13, 14, 52). In brief, trypsinized MC38, CMT93, and Pan02 cells were resuspended in cold phosphate-buffered saline. The mice, having been fed either an LFD or an HFD, were subjected to anesthesia. After a laparotomy, MC38 cells (2 × 10^4^ or 2 × 10^5^ cells), MC38 cells stably expressing either shCon or sh*Yap1* (1 × 10^5^ cells), CMT93 cells (1 × 10^6^ cells), and Pan02 cells (1 × 10^6^ cells) were injected into the spleen. The spleen was removed, and the abdomen was closed. After 2 weeks of cancer cell inoculation, mice were euthanized for subsequent analysis and sampling. For the prevention study with HA synthesis inhibitors, mice were divided into 6 groups: LFD-vehicle (*n* = 8), LFD–4-methylumbelliferone (4-MU) (*n* = 6), LFD–4-methylumbelliferyl-β-d(–)-glucuronide (4-MUG) (*n* = 8), HFD-vehicle (*n* = 8), HFD–4-MU (*n* = 9), and HFD–4-MUG (*n* = 9). 450 mg/kg of 4-MU (Sigma-Aldrich, catalog M1508) or vehicle control (2% sucrose) was orally administered daily, and 4-MUG (Chem-Impex, catalog 20981) was administered to mice via drinking water at a final concentration of 2 mg/mL, starting 2 weeks before the injection of cancer cells and continued until the point of sacrifice. For the treatment study, mice were treated with 4-MU (orally administered at 450 mg/kg daily), starting 1 week after tumor inoculations (2 × 10^4^ MC38 cells) and continued for an additional 3 weeks. For the survival study, mice were treated with 4-MU (orally administered at 450 mg/kg daily), starting 1 week after tumor inoculations (2 × 10^4^ MC38 cells) and continuing until death. For the combination therapy, mice were treated with 4-MU (orally administered at 450 mg/kg daily) and anti–PD-1 antibody (Bio X Cell, catalog BP0146; 200 μg intraperitoneally every 3 days) or control IgG (Bio X Cell, catalog BP0089) 1 day after MC38 cell inoculation (2 × 10^5^ cells) and continuing for an additional 2 weeks.

### Statistics.

Statistical significance was confirmed by GraphPad Prism 8 (GraphPad Software Inc.). Statistical significance was compared between the 2 groups by 2-tailed unpaired Student’s *t* test. Comparisons between different groups were performed using 1-way ANOVA test, followed by Tukey’s post hoc analysis. Correlation analysis was performed using Pearson’s correlation coefficient. Survival curves were generated using the Kaplan-Meier method and compared using the log-rank test. A *P* value less than 0.05 was considered significant.

### Study approval.

Samples from patients with CRC liver metastasis were analyzed. The study was approved by the Cedars-Sinai Medical Center Institutional Review Board (IRB no. 0901). Written informed consent was obtained from all participants. All animal experiments were approved by the Cedars-Sinai Medical Center Institutional Animal Care and Use Committee (IACUC no. 8412) and the Kangwon National University Animal Protection and Use Committee (IACUC nos. KW-200225-1 and KW-240620-1).

### Data availability.

RNA-Seq data of the liver metastatic tumor tissue in HFD-fed and LFD-fed mice and the gene expression profile at the single-cell level of immune cells from the liver-metastasized tumor tissue were previously deposited in the NCBI’s Gene Expression Omnibus database (GEO GSE227913 and GSE227914). All supporting data are provided in the Supporting Data Values file.

## Author contributions

ES, YMY, SY, and AMX designed research studies. YMY, ZW, SYK, Jieun Kim, GJC, JHL, SMK, AMX, AM, SY, and Jina Kim conducted experiments. YMY, ZW, Jieun Kim, SYK, GJC, JHL, SMK, TT, and MM acquired data. YMY, ZW, SYK, Jieun Kim, GJC, AMX, Jina Kim, SY, and ES analyzed data. ES and YMY wrote the original draft. YMY, AMX, Jieun Kim, VP, SJP, NAB, SY, SCL, and ES reviewed and edited the manuscript. ES, EMP, NAB, SCL, Jieun Kim, and YMY acquired funding. AG, MSL, AM, PWN, DJ, and EMP provided resources. ES supervised the study. The order in which the co–first authors are listed was determined by the order of their entry into the study.

## Supplementary Material

Supplemental data

Unedited blot and gel images

Supporting data values

## Version 1 02/13/2025

In-Press Preview

## Version 2 04/01/2025

Electronic publication
