# Supplementary material for: Metastatic tumor growth in steatotic liver is promoted by HAS2-mediated fibrotic tumor microenvironment
Source: J Clin Invest. 2025 Feb 13;135(7):e180802. doi: 10.1172/JCI180802 (PMC11957696; doi:10.1172/JCI180802)
Supplement: Supplemental data [file jci-135-180802-s028.pdf]

Supplemental Materials

Metastatic Tumor Growth in Steatotic Liver is Promoted by HAS2-Mediated Fibrotic Tumor Microenvironment

Yoon Mee Yang[1,2,3,4#], Jieun Kim[1,#], Zhijun Wang[1,5,##], Jina Kim[6,7,8,##], So Yeon Kim[1], Gyu Jeong Cho[2,4], Jee Hyung Lee[2], Sun Myoung Kim[2,4], Takashi Tsuchiya[1], Michitaka Matsuda[1], Vijay Pandyarajan[1], Stephen J. Pandol[1], Michael S. Lewis[9,10], Alexandra Gangi[11], Paul W. Noble[12], Dianhua Jiang[12], Akil Merchant[6,13], Edwin M. Posada[6,14], Neil A. Bhowmick[6,14], Shelly C. Lu[1], Sungyong You[6,7,8,*], Alexander M. Xu[6,15,16,*], Ekihiro Seki[1,6,15,*]

[1]Karsh Division of Gastroenterology and Hepatology, Department of Medicine, Cedars-Sinai Medical Center, Los Angeles, CA 90048, USA, [2]Department of Pharmacy, Kangwon National University, Chuncheon 24341, South Korea, [3]Multidimensional Genomics Research Center, Kangwon National University, Chuncheon 24341, Republic of Korea, [4]Innovative Drug Development Research Team for Intractable Diseases (BK21 plus), Kangwon National University, Chuncheon, 24341, Republic of Korea, [5]Division of Gastroenterology, Union Hospital, Tongji Medical College, Huazhong University of Science and Technology, Wuhan, China, [6]Samuel Oschin Comprehensive Cancer Institute, Cedars-Sinai Medical Center, Los Angeles, CA 90048, USA, [7]Department of Urology, Cedars-Sinai Medical Center, Los Angeles, CA 90048, USA, [8]Department of Computational Biomedicine, Cedars-Sinai Medical Center, Los Angeles, CA 90048, USA, [9]Department of Pathology, Cedars-Sinai Medical Center, Los Angeles, CA 90048, USA, [10]Department of Pathology, Veterans Affairs Greater Los Angeles Health Care System, Los Angeles, CA 90073, USA, [11]Department of Surgery, Cedars-Sinai Medical Center, Los Angeles, CA 90048, USA, [12]Department of Medicine and Women's Guild Lung Institute, Cedars-Sinai Medical Center, Los Angeles, CA 90048,USA, [13]Division of Hematology and Cellular Therapy, Department of Medicine, Cedars-Sinai Medical Center, Los Angeles, CA 90048, USA, [14]Division of Medical Oncology, Department of Medicine, Cedars-Sinai Medical Center, Los Angeles, CA 90048, USA, [15]Department of Biomedical Sciences, Cedars-Sinai Medical Center, Los Angeles, CA 90048, USA, [16]Fischell Department of Bioengineering, A. James Clark School of Engineering, University of Maryland, College Park, MD 20742, USA

# YMY and Jieun K are the co-first authors and contributed equally to this work.
ZW and Jina K contributed equally as the second authors of this work.

***Correspondence to:**
Ekihiro Seki, M.D., Ph.D.
Karsh Division of Gastroenterology and Hepatology, Department of Medicine,
Cedars-Sinai Medical Center
8700 Beverly Blvd., Davis Research Bldg., Suite 2099, Los Angeles, CA 90048
Phone: +1-310-423-6605
Fax: +1-310-423-0157
E-mail: Ekihiro.Seki@cshs.org

Alexander M. Xu, PhD
Fischell Department of Bioengineering, A. James Clark School of Engineering,
University of Maryland
8728 Paint Branch Drive, College Park, MD 20742
E-mail: alexmxu@umd.edu

Sungyong You, PhD
Department of Urology and Computational Medicine
Cedars-Sinai Medical Center
8700 Beverly Blvd., Davis Research Bldg., Suite 2099, Los Angeles, CA 90048
E-mail: Sungyong.You@cshs.org

The authors have declared that no conflict of interest exists.

Supplemental Methods

Tumor analysis

Metastatic liver tumors were examined both through macroscopic and histological analysis. Photographs of the liver with tumors were taken to verify the macroscopic features. The number of tumor nodules was counted, and the maximum tumor diameter and liver weight were measured. Tumor areas were quantified from H&E-stained liver sections utilizing the National Institutes of Health ImageJ software program.

Histological analysis

Mouse liver tissues were fixed with 10% formalin overnight at room temperature and embedded with paraffin. For patients' samples, tissue microarrays (TMAs) containing formalin-fixed, paraffin-embedded sections of primary CRC, CRC liver metastases, and adjacent non-tumorous liver tissues were utilized. The embedded paraffin blocks were cut to a thickness of 4 μm. After deparaffinization and rehydration, hematoxylin and eosin (H&E, Leica, Cat# 3801698) and Sirius red (Abcam, Cat# ab246832) staining was performed according to the manufacturer's instruction. For immunohistochemistry staining, avidin/biotin technology was employed. Prior to immunohistochemistry procedures, target retrieval solution (Citrate pH 6.1, Dako, Cat# S169984-2) was used for antigen retrieval. The sections were incubated with primary antibodies, including mouse

monoclonal anti-alpha smooth muscle actin (α-SMA) antibody (Dako, Cat# M085129-2), rat monoclonal anti-F4/80 monoclonal antibody (eBioscience, Cat# 14-4801), mouse monoclonal anti-YAP antibody (Santa Cruz Biotechnology, Cat# sc-101199), mouse monoclonal anti-CTGF antibody (Santa Cruz Biotechnology, Cat# sc-365970), or goat polyclonal anti-mouse CD206 antibody (R&D Systems, Cat# AF2535). When mouse primary antibodies were applied on the mouse tissue sections, the M.O.M. Kit (Vector Laboratories, Cat# PK-2200) was used to reduce non-specific background staining according to the manufacturer's instruction. For HA staining, liver sections were incubated with 2 μg/ml of biotin-labeled HA binding protein (rhAggrecan aa20-675/His biotin, R&D systems). Then, the sections were developed using VECTASTAIN Elite ABC Kit (Vector Laboratories, Cat# PK-6100) and DAB Peroxidase Substrate Kit (Vector Laboratories, Cat# SK-4100). For oil red O staining, frozen liver sections were submerged in the oil red O working solution dissolved in isopropanol. Slides were incubated in the staining solution for 30 minutes at room temperature. The stained slides were thoroughly rinsed with distilled water, and a mounting medium was applied. Images were captured using a DMi8 inverted microscope (Leica) at Cedars-Sinai Medical Center and an Axio Imager A2 microscope (Zeiss) or an ImageXpress Pico (Molecular Devices) at the Core-Facility for Innovative Cancer Drug Discovery (CFICDD) at Kangwon National University. Image analysis was performed by ImageJ software. The list of antibodies used in this study is provided in Supplemental Table 2.

Immunofluorescence staining

After sacrifice, the liver tissues were fixed with 4% buffered formalin solution, and then dehydrated with 10% and 30% sucrose-phosphate-buffered saline solution. Then, tissues were mounted in OCT embedding compound, and subsequently frozen at -80℃. Tissues were sectioned at a thickness of 10 μm using a cryostat. The cryostat sections were thawed at room temperature for 10-20 minutes. The slides were rehydrated in PBS solution. The sections were incubated with primary antibodies, including mouse monoclonal anti-alpha smooth muscle actin (α-SMA) antibody (Dako, Cat# M085129-2) or rat monoclonal anti-CD44 antibody (BD Biosciences, Cat# 553131) or rabbit polyclonal anti-Desmin (Epredia, Cat# RB9014P0). For α-SMA staining, the M.O.M. Kit (Vector Laboratories, Cat# PK-2200) were used to reduce non-specific background staining according to the manufacturer's protocol. The donkey anti-mouse IgG (H+L) secondary antibody, Alexa Fluor 488 conjugated (Invitrogen, Cat# A21202), donkey anti-rat IgG (H+L) secondary antibody, or Alexa Fluor 488 conjugated (Invitrogen, Cat#A21208) were applied. Finally, SlowFade^{TM} Diamond Antifade Mountant with DAPI (ThermoFisher Scientific, Cat# S36968) were added to the samples. Images were captured by a Zeiss LSM800 microscope or a BZ-X710 Keyence fluorescence microscope.

RNAscope

Has2 RNA *in situ* hybridization assay was performed on formalin-fixed, paraffin-embedded tissue

sections using RNAscope® ACD HybEZ II Hybridization System with ACD EZ-Batch Slide system (Advance Cell Diagnostics; ACD) and RNAscope Multiplex Fluorescent Reagent Kit v2 (ACD; Cat# 323110) according to the manufacturer's instructions. The mouse *Has2*-specific RNAscope probe (ACD; Cat# 465171) were hybridized for 2 hours at 40 °C. Signal visualization was performed by Opal dyes, Opal 570 Reagent (Akoya Biosciences, FP1488001KT) or Opal 690 Reagent (Akoya Biosciences, FP1497001KT). When co-stained with primary antibodies against α-SMA, F4/80, and CD31 (Abcam, Cat# ab28364), tissue sections were incubated overnight at 4 °C in primary antibodies in solution with Co-Detection Diluent and then incubated with Alexa Fluor 488 conjugated, secondary antibody solutions for 1 hour at room temperature. Images were captured by a BZ-X710 Keyence fluorescence microscope.

Quantitative real-time polymerase chain reaction (qRT-PCR)

The tissues were minced using a Minilys homogenizer (Bertin Technologies) with TRIzol Reagent (Thermo Fisher Scientific, Cat# 15596018), and total RNA was subsequently extracted utilizing the AccuPrep RNA Extraction Kit (Bioneer, Cat# K-3140). It was reverse transcribed into complementary DNA (cDNA) using High-Capacity cDNA Reverse Transcription Kit (Thermo Fisher Scientific, Cat# 4368814). ExcelTaq™ 2X Fast Q-PCR Master Mix (SYBR, SMOBIO, Cat# TQ1210)-based qPCR analysis was conducted using CFX96 Real-Time PCR system (Bio-Rad). The sequence of primers used

in this study is listed in Supplemental Table 3.

Serum and tissue HA measurement

Blood was collected via orbital blood sampling. Subsequently, the blood samples were allowed to stand at room temperature for 30 minutes, and serum was extracted through centrifugation. The obtained serum was stored in -80℃ deep freezer until use. The metastatic liver tumor tissues were homogenized in PBS, incubated with Pronase E (0.5 mg/mL) for 3 hours at 60 °C, and then heat-inactivated for 30 minutes at 100 °C. Supernatant was collected after centrifugation for further analysis. Measurement of HA levels in the serum and tissues was performed using a Hyaluronan DuoSet ELISA Kit (R&D Systems, Cat# DY3614), according to manufacturer's protocol. To measure the size-specific HA fraction levels in the serum and tissue, 100- and 300-kDa cutoff columns (Centrisart, Sartorius, Cat# 13269-E and 13279-E) were employed, as previously described(1). The absorption was read at 450nm and 570nm using SpectraMax i3 (Molecular Devices).

In vitro HA treatment

Low molecular weight hyaluronan (15-40 kDa) and high molecular weight hyaluronan (>950 kDa) were purchased from R&D Systems. Serum-deprived MC38 cells were treated with 100 μg/ml of HA for 16-24 hours.

Colony formation assay

Sterile solution of 1.6% base low-molecular weight (LMW) agar were prepared in deionized water. Subsequently, a mixture of 1.6% LMW agar and 2× DMEM with 20% FBS at a 1:1 ratio was added to each well of a 6-well plate (2 mL per well). Once the base agar solidified, 0.4% top agar containing 10% FBS and 100 μg/ml HA was added to the plates along with the cells. The final concentration of cells in each well was adjusted to 2×10^3 cells/well. The plates were placed on a flat surface at 4°C for 15 minutes to solidify the mixture. Cells were fed 1-2 times per week with cell culture media containing 10% FBS and 100 μg/ml HA. Following 2–3 weeks of incubation at 37°C and 5% CO_2, the cell colonies were fixed with 4% paraformaldehyde for 10 minutes and stained with 0.05% crystal violet solution. Colonies with a diameter >1 mm were quantified using ImageJ software(2).

Invasion assay

MC38 cells were deprived of serum overnight and then treated with 100 μg/ml HA for 16-24 h. Cells were harvested and resuspended in serum-free DMEM. 1×10^4 cells were seeded in BioCoat Matrigel Invasion Chambers with 8.0 μm PET Membrane (Corning, Cat# 354480), and DMEM with 10% FBS was loaded into the lower chamber. After 16-24 hours of seeding, cells were washed with PBS, fixed in 4% paraformaldehyde, and stained with H&E. For each independent experiment, four inserts per

condition were used. Eight fields were randomly chosen under a 10× objective lens on a DMi8 inverted microscope (Leica). Quantification of the number of invading cells in each image was performed using the “Analyze Particles” function of ImageJ(2).

Primary cell isolation and treatment

Hepatic stellate cells (HSCs) and Kupffer cells were isolated from mice through in situ liver perfusion with Pronase E (Sigma-Aldrich, Cat# 10165921001) and Collagenase D (Sigma-Aldrich, Cat# 11088882001) followed by density gradient centrifugation using Nycodenz (Accurate Chemical and Scientific Corporation, Cat# AN1002424), as previously detailed(1). The methods for hepatocyte isolations were described previously(1). Magnetic antibody sorting (MACS; Miltenyi biotec) employing CD11b (Miltenyi Biotec, Cat# 130-097-142) was used to deplete contaminating $CD11b^+$ cells in the HSC fraction. The purity of isolated HSCs was assessed based on autofluorescence of Vitamin A under a LEICA DMi8 fluorescent microscope. In the in vitro CTGF or CYR61 treatment experiment, primary HSCs were treated with 20 ng/ml of recombinant CTGF (R&D System, Cat# 9190-CC) or 10 ng/ml of CYR61(ABclonal, Cat# RP00353) for a duration of 24 hours.

Co-culture

Primary HSCs were cultured in 24-well plates with medium containing 10% FBS for 5 days.

Subsequently, HSCs were maintained overnight in serum-free medium before the initiation of co-culture experiments. MC38 cells, stably transfected with either shCon or sh*Yap1*, were cultured on 0.4-μm pore size cell culture inserts (BD Biosciences) at a density of 1×10^4 cells/well for 12 hours and then maintained in serum-free medium for overnight before co-culture. For transient transfection of small interfering RNA (siRNA), MC38 cells were transfected with either siCon (Santa Cruz Biotechnology, Cat# sc-39330) or si*Ccn2* (Santa Cruz Biotechnology, Cat# sc-39330) using Lipofectamine RNAiMAX Transfection Reagent (Cat# 13778150, Invitrogen) for 36 hours before co-culture. The inserts were then placed in the plate for a 48-hour co-culture period. Independent culture experiments were conducted, with three replicates.

FAK inhibitor treatment

For immunocytochemistry experiment, MC38 cells were seeded onto coverslips and maintained in serum-free medium overnight. The cells were treated with 4 μM PF-562271 (FAK inhibitor, Selleckchem, Cat# S2890) for 30 minutes, followed by 0.5 μg/mL HA for 2 hours. Cells were then fixed with 4% paraformaldehyde, permeabilized with PBS containing 0.25% Triton X-100, and blocked for 1 hour at room temperature. After blocking, cells were incubated with the primary antibody against YAP1 overnight at 4°C, followed by Alexa Fluor 594-conjugated secondary antibody for 1 hour at room temperature in the dark. Nuclei were counterstained with DAPI, and coverslips were mounted

for imaging. For western blot analysis, MC38 cells were seeded in a culture plate for 12 hours and incubated in serum-free medium overnight. Following serum starvation, the cells were treated with 4 μM PF-562271 for 30 minutes and subsequently with 0.5 μg/mL HA for 12 hours. After the treatment period, protein lysates were collected for Western blot analysis.

Western blots

Cell and tissue samples were homogenized using a RIPA buffer supplemented with a protease inhibitor cocktail (Millipore, Cat# 535140) and phosphatase inhibitor cocktail 2 (Sigma-Aldrich, Cat# P5726), followed by incubation on ice for 30 minutes. After centrifugation, total protein lysates were obtained. Cytosolic and nuclear proteins were extracted from MC38 cells using NE-PER™ Nuclear and Cytoplasmic Extraction Reagents, following the manufacturer's protocol (Thermo Fisher Scientific, Cat# 78835). Then, Bradford protein assay was performed at room temperature to determine the protein concentration using Pierce Bradford Protein Assay Kit (Thermo Scientific, Cat# 23200). The samples were subjected to protein denaturation by heating the samples at 95-100°C for 5 minutes. Equal amounts of protein were loaded onto an SDS-Page gel, and then proteins were transferred from the gel to a nitrocellulose membrane. The membrane was blocked with 5% skim milk in TBS-T (Tris-buffered saline with 0.1% Tween-20) for 1 hour at room temperature. Primary antibodies against phospho-YAP (Ser127) (Cell Signaling Technology, Cat# 4911), YAP (Santa Cruz Biotechnology, Cat# sc-101199),

Collagen type I (Millipore, Cat# AB765P), α-SMA (Abcam, Cat# ab7817), α-tubulin (Sigma-Aldrich, Cat# T5168), Lamin B1 (Santa Cruz Biotechnology, Cat# sc-374015), β-actin (Sigma-Aldrich, Cat# A5441), and GAPDH (Santa Cruz Biotechnology, Cat# sc-32233) were incubated overnight at 4°C. After washing, HRP-conjugated secondary antibodies were applied for 1 hour at room temperature. To visualize protein bands, an enhanced chemiluminescence (ECL) substrate for HRP detection was utilized and detected by the FUSION SOLO X with the eVo-6 camera (Vilber).

Bulk RNA sequencing analysis

Bulk RNA sequencing was performed by BGI using tumor samples obtained from four experimental groups: LFD-fed mice with liver metastatic tumors, HFD-fed mice with liver metastatic tumors, HFD-fed mice with liver metastatic tumors induced by the splenic injection of shCon-MC38 cells, and HFD-fed mice with liver metastatic tumors induced by the splenic injection of shYap-MC38 cells(2). The sequencing data deposited in gene expression omnibus (GSE227913) were used(2). Sequencing reads were aligned to the reference mouse genome (GRCm38/mm10) using STAR (version 2.7.3a) through Partek Flow software. For differential gene expression analysis with raw read counts, DESeq2 was employed. The p-values from multiple tests were adjusted using the Benjamini-Hochberg method, and significance was determined at a threshold of false discovery rate (FDR)<0.01 and $|\log_2$ (fold change)$| > 1$. Gene set enrichment analysis (GSEA) was performed using the GSEA software (version

4.3.2, https://www.gsea-msigdb.org/gsea/index.jsp). Normalized enrichment score, p-value, and FDR value were produced by 1000 permutations, and these values were shown in the graphs.

Single-cell RNA Sequencing

The sequencing data that we previously deposited to NCBI Gene Expression Omnibus (GSE227914) were used(2). The demultiplexed raw reads were aligned to the transcriptome using STAR (version 2.5.1) with default parameters, using mouse mm10 transcriptome reference from Ensembl version 84 annotation, containing all protein coding and long non-coding RNA genes. Expression counts for each gene in all samples were collapsed and normalized to unique molecular identifier (UMI) counts using Cell Ranger software version 4.0.0 (10x Genomics). The processed data contains a large digital expression matrix with cell barcodes as rows and gene identities as columns. The processed matrix were loaded into the R with the Seurat package v4.0.5 in accordance with the introductory vignettes workflow by Seurat using their default parameters(3). Outlier cells were filtered using library size, number of expressed genes, and mitochondrial proportion (nFeature_RNA > 200, nFeature_RNA < 7500, and percent.mt < 25). The read counts from Cell Ranger were normalized and log_2 transformed using the NormalizeData function of the Seurat package. Samples were integrated, and principal component analysis was performed by 'RunPCA', followed by 'RunUMAP' for dimensionality reduction. The first 20 principal components were used for clustering, with 1.0 set as the resolution

parameter. Some clusters were divided by gene expression for clarification of clustering. Differential gene expression was assessed using the 'FindAllMarkers' function of the Seurat default function. The cell-cell interactions were analyzed and visualized using the R package CellChat(4).

Imaging Mass Cytometry Analysis

Raw IMC data were processed to obtain single-cell masks (segmentation) and average protein expression per cell using the IMC Segmentation pipeline (https://github.com/BodenmillerGroup/ImcSegmentationPipeline). Briefly, cell segmentation was performed using Ilastik. Select channels were chosen for Ilastik training to identify nuclei (DNA1/DNA2/HistoneH3), cell cytosol (αSMA/CK19/HLAABC/HLADR/HepPar1/CD68), and background areas. Average protein and morphological measurements were taken for each cell using Python and CellProfiler.

Further IMC processing was performed using R packages. Using the output from the IMC Segmentation pipeline, cells larger than 1000 pixels (μm^2) and smaller than 15 pixels were removed. Images with fewer than 500 cells and patient samples with fewer than 5,000 cells were also removed. Single-cell protein measurements were arcsinh transformed using a co-factor of 5 and censored at the 99th percentile. Cell phenotyping was performed using PhenoGraph and plotted using UMAP. Two levels of PhenoGraph were performed using cell phenotyping and functional proteins to obtain major

cell types (immune, liver, CAFs, endothelial, and cancer cells) and immune-functional subtypes by manual curation of PhenoGraph clusters. Cell density of type A relative to a cell of type B was defined as the difference between 1 and the mean distance of the five nearest A cells (censored at 100 μm) from a B cell, divided by 100 μm. Thus, density ranged from 0 (no A cells within 100 μm) to ~1. Spatial regions with high liver-cell density were identified and excluded from analysis to obtain metastatic tumor regions from liver metastasis tissue samples. A cell was defined as being in a high liver-density zone if its liver density was greater than 0.7 (mean distance of 5 nearest liver cells < 30 μm. Single-cell measurements and patient-level measurements were compared using two-tailed Student's *t*-test or Mann-Whitney *U* test or generalized linear models (GLM) using the sample as a clustering variable to obtain robust standard error.

Supplemental References

1. Yang YM, Noureddin M, Liu C, Ohashi K, Kim SY, Ramnath D, et al. Hyaluronan synthase 2-mediated hyaluronan production mediates Notch1 activation and liver fibrosis. *Sci Transl Med.* 2019;11(496).
2. Wang Z, Kim SY, Tu W, Kim J, Xu A, Yang YM, et al. Extracellular vesicles in fatty liver promote a metastatic tumor microenvironment. *Cell Metab.* 2023;35(7):1209-26 e13.
3. Butler A, Hoffman P, Smibert P, Papalexi E, and Satija R. Integrating single-cell transcriptomic data across different conditions, technologies, and species. *Nat Biotechnol.* 2018;36(5):411-20.
4. Jin S, Guerrero-Juarez CF, Zhang L, Chang I, Ramos R, Kuan CH, et al. Inference and analysis of cell-cell communication using CellChat. *Nat Commun.* 2021;12(1):1088.

Supplemental Table 1. Patient Demographics for Tissue Microarray and Imaging Mass Cytometry in Colorectal Cancer Liver Metastasis Study

Characteristics	Patients with CRC liver metastasis and concurrent MASLD (n = 13)	Patients with CRC liver metastasis without concurrent MASLD (n = 17)	*P*-value
**Age, median (range)**	59 (38-71)	55 (34-89)	0.421
**BMI (range)**	30.68 (19.55-39.76)	23.77 (19.40-34.14)	**0.0042**
**Steatosis**			
Score 0 (<5%)	0 (0%)	17 (100.0%)	**<0.0001**
Score 1 (5-33%)	8 (61.5%)	0 (0%)	
Score 2 (34-66%)	1 (7.7%)	0 (0%)	
Score 3 (>66%)	4 (30.8%)	0 (0%)	
**Gender, n (%)**			
Female	7 (53.8%)	6 (35.3%)	0.4601
Male	6 (46.2%)	11 (64.7%)	
**Race, n (%)**			
Caucasian	6 (46.2%)	8 (47.1%)	0.3604
African-American	2 (15.4%)	0 (0%)	
Asian	1 (7.7%)	3 (17.6%)	
Other	4 (30.8%)	6 (35.3%)	
**Chemotherapy, n (%)**			
No	2 (15.4%)	4 (23.5%)	0.6725
Yes	11 (84.6%)	13 (76.5%)	

Supplemental Table 2. List of antibodies used in this study.

Name	Citation	Supplier	Catalog no.	Clone no.
Mouse Monoclonal Anti-α-SMA	RRID:AB_2811108	Dako	M085129-2	1A4
Mouse Monoclonal Anti-α-SMA	RRID:AB_262054	Abcam	Ab7817	1A4
Mouse Monoclonal Anti-YAP	RRID:AB_1131430	Santa Cruz Biotechnology	sc-101199	63.7
Rat Monoclonal Anti-F4/80	RRID:AB_467559	Thermo Fisher Scientific	14-4801-85	BM8
Goat Polyclonal Anti-CD206	RRID:AB_2063012	R&D Systems	AF2535	N/A
Rat Monoclonal Anti-CD44	RRID:AB_394646	BD Biosciences	553131	IM7
Rabbit Polyclonal Anti-Collagen Type I	RRID:AB_92259	Millipore	AB765P	N/A
Rabbit Polyclonal Anti-Phospho-YAP (Ser127)	RRID:AB_2218913	Cell Signaling Technology	4911	N/A
Mouse Monoclonal Anti-CTGF	RRID:AB_10917259	Santa Cruz Biotechnology	sc-365970	E-5
Rabbit Polyclonal Anti-CD31	RRID:AB_726362	Abcam	ab28364	N/A
Rabbit Polyclonal Anti-Desmin	RRID:AB_149768	Epredia	RB9014P0	D33
Mouse Monoclonal Anti-GAPDH	RRID:AB_627679	Santa Cruz Biotechnology	sc-32233	6C5
Mouse Monoclonal Anti-α-tubulin	RRID:AB_477579	Sigma-Aldrich	T5168	B-5-1-2
Mouse Monoclonal Anti-β-actin	RRID:AB_476744	Sigma-Aldrich	A5441	AC-15
Mouse Monoclonal Anti-Lamin B	RRID:AB_10947408	Santa Cruz Biotechnology	sc-374015	N/A
Donkey Anti-Mouse IgG (H+L) Antibody, Alexa Fluor 488 Conjugated	RRID:AB_141607	Thermo Fisher Scientific	A-21202	N/A
Donkey Anti-Rat IgG (H+L) Antibody, Alexa Fluor 488 Conjugated	RRID:AB_2535794	Thermo Fisher Scientific	A-21208	N/A
Donkey Anti-Rabbit IgG (H+L) Antibody, Alexa Fluor 488 Conjugated	RRID:AB_2535792	Thermo Fisher Scientific	A-21206	N/A
OPAL™ 570 Reagent Pack	RRID:N/A	Akoya Biosciences	FP1488001KT	N/A
OPAL™ 690 Reagent Pack	RRID:N/A	Akoya Biosciences	FP1497001KT	N/A
Goat anti-Rabbit IgG (H+L) Antibody, HRP-linked	RRID:AB_228341	Thermo Fisher Scientific	31460	N/A
Goat anti-Mouse IgG (H+L), HRP-linked	RRID:AB_228307	Thermo Fisher Scientific	31430	N/A
Biotin Goat Anti-Mouse IgG	RRID:AB_395196	BD Biosciences	553999	N/A
Biotin Mouse Anti-Rat IgG	RRID:AB_466659	Thermo Fisher Scientific	13-4813-85	N/A
Biotin Mouse Anti-Goat IgG	RRID:AB_228368	Thermo Fisher Scientific	31730	N/A
Mouse Monoclonal Anti-CD31	RRID:AB_2756834	Abcam	ab187377	C31.3
Rabbit Monoclonal Anti-CDX2	RRID:N/A	Novus Biologicals	NBP3-08738	2951R
Mouse Monoclonal Anti-CD11c	RRID:N/A	Abcam	ab212508	IGTAX/1242
Rabbit Monoclonal Anti-YAP1	RRID:AB_2219141	Abcam	ab52771	EP1674Y

Mouse Monoclonal Anti-CK19	RRID:AB_307088	Abcam	ab9221	RCK108
Mouse Monoclonal Anti-Galectin-9	RRID:N/A	Millipore	MABT833	1G3
Rabbit Monoclonal Anti-PD-L1	RRID:AB_2687878	Abcam	ab205921	28-8
Sheep Polyclonal Anti-FAP	RRID:AB_2102369	R&D Systems	AF3715	N/A
Rabbit Monoclonal Anti-CD11b	RRID:AB_2915959	Abcam	ab209970	EPR1344
Mouse Monoclonal Anti-HepPar1	RRID:N/A	NSJ Bioreagents	V3109SAF	V3109
Mouse Monoclonal Anti-CD4	RRID:N/A	Fluidigm	3156036D	EPR6855
Mouse Monoclonal Anti-CD68	RRID:AB_2810859	Fluidigm	3159035D	KP1
Rabbit Monoclonal Anti-VISTA	RRID:AB_2811070	Fluidigm	3160025D	D1L2G
Mouse Monoclonal Anti-CD8a	RRID:AB_2811053	Fluidigm	3162034D	C8/144B
Rabbit Monoclonal Anti-TIM3	RRID:AB_2716862	Cell Signaling Technology	45208	D5D5R™
Mouse Monoclonal Anti-PD1	RRID:N/A	Abcam	ab201811	NAT105
Mouse Monoclonal Anti-Ki-67	RRID:AB_396287	BD Biosciences	556003	B56
Rabbit Polyclonal Anti-CD3	RRID:AB_2811048	Fluidigm	3170019D	N/A
Rat Monoclonal Anti- CD44	RRID:AB_2895121	Fluidigm	3171003B	IM7
Mouse Monoclonal Anti-αSMA	RRID:N/A	Novus Biologicals	NBP2-34522	1A4/asm-1
Rabbit Monoclonal Anti-Histone H3	RRID:N/A	Abcam	ab238971	EPR16987
Rabbit Monoclonal Anti-CD45	RRID:AB_2922773	Cell Signaling Technology	47937	D9M8I

Supplemental Table 3. Primer pairs used for quantitative reverse-transcription polymerase chain reaction analysis

Gene	Forward (5′ to 3′)	Reverse (5′ to 3′)
18S rRNA	AGTCCCTGCCCTTTGTACACA	CGATCCGAGGGCCTCACTA
Acta2	ACTGGGACGACATGGAAAAG	GTTCAGTGGTGCCTCTGTCA
Ankrd1	GGAACAACGGAAAAGCGAGAA	GAAACCTCGGCACATCCACA
Axl	TGGTGAGGGAGGAGCATGTT	AAAAGAAGGGGAGCTTGCTGA
Ccl2	CCTGCTGTTCACAGTTGCC	ATTGGGATCATCTTGCTGGT
Ccn1	TAAGGTCTGCGCTAAACAACTC	CAGATCCCTTTCAGAGCGGT
Ccn2	GGGCCTCTTCTGCGATTTC	ATCCAGGCAAGTGCATTGGTA
Cd44	GCACTGTGACTCATGGATCC	TTCTGGAATCTGAGGTCTCC
Col1a1	CCTCAGGGTATTGCTGGACAAC	CAGAAGGACCTTGTTTGCCAGG
Has1	CTATGCTACCAAGTATACCTCG	TCTCGGAAGTAAGATTTGGAC
Has2	TGAGCAGGAGCTGAACAAGA	GCCAACAATATAAGCAGCTGTG
Has3	GATGTCCAAATCCTCAACAAG	CCCACTAATACATTGCACAC
Il1b	GGTCAAAGGTTTGGAAGCAG	TGTGAAATGCCACCTTTTGA
Il4	GGTCTCAACCCCCAGCTAGT	GCCGATGATCTCTCTCAAGTGAT
Il13	CACACAAGACCAGACTCCCC	TCTGGGTCCTGTAGATGGCAAT
Mrc1	CAGGTGTGGGCTCAGGTAGT	TGTGGTGAGCTGAAAGGTGA
Nos2	ACATCGACCCGTCCACAGTAT	CAGAGGGGTAGGCTTGTCTC
Timp1	AGGTGGTCTCGTTGATTTCT	GTAAGGCCTGTAGCTGTGCC
Tlr4	TGTTCTTCTCCTGCCTGACA	TGTCATCAGGGACTTTGCTG
Yap1	ACCCTCGTTTTGCCATGAAC	TGTGCTGGGATTGATATTCCGTA

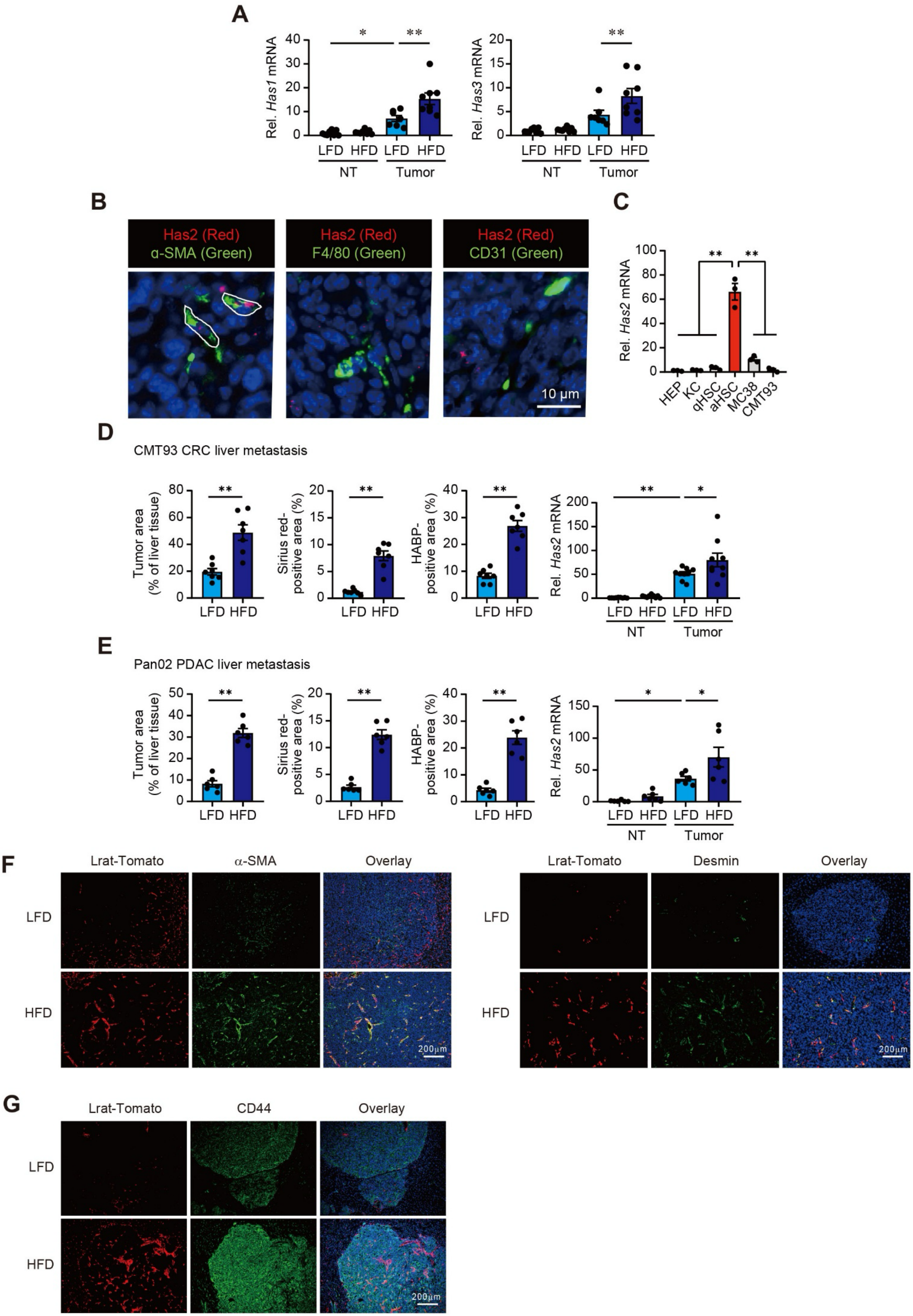
A
Rel. Has1 mRNA
Rel. Has3 mRNA
LFD HFD LFD HFD
NT Tumor
B
Has2 (Red) α-SMA (Green)
Has2 (Red) F4/80 (Green)
Has2 (Red) CD31 (Green)
10 μm
C
Rel. Has2 mRNA
HEP KC qHSC aHSC MC38 CMT93
D
CMT93 CRC liver metastasis
Tumor area (% of liver tissue)
Sirius red-positive area (%)
HABP-positive area (%)
Rel. Has2 mRNA
E
Pan02 PDAC liver metastasis
F
Lrat-Tomato α-SMA Overlay
Lrat-Tomato Desmin Overlay
LFD
HFD
200μm
G
Lrat-Tomato CD44 Overlay
LFD
HFD
200μm

Supplemental Figure 1. Hepatic stellate cells are responsible for hyaluronan synthase 2 expression in tumors.

(A) Comparison of mRNA expression levels of *Has1* and *Has3* in nontumor (NT) and tumor tissues from mice on a low-fat diet (LFD) or a high-fat diet (HFD). (n = 7-8 per group)
(B) Representative images of RNAscope in situ hybridization showing *Has2* expression (red), co-stained with α-SMA, F4/80, or CD31 antibodies (green) to visualize activated hepatic stellate cells (HSCs), macrophages, and endothelial cells, respectively.
(C) Comparison of *Has2* mRNA expression levels among primary hepatocytes (HEP), Kupffer cells (KC), quiescent HSCs (qHSC), activated HSCs (aHSC), MC38, and CMT93 cells. Relative expression levels were normalized to those of HEP and presented accordingly. (n = 3)
(D) CMT93 colorectal cancer (CRC) liver metastasis model. Mice were fed either a LFD or an HFD for 6 weeks, followed by spleen injection of CMT93 cells. Diet feeding was continued for an additional 2 weeks before the experiment was completed. The figure presents data on tumor area, Sirius red-positive area (%), HABP-positive area (%) (n = 7), and *Has2* mRNA expression levels. (n = 9)
(E) Pan02 pancreatic ductal adenocarcinoma (PDAC) liver metastasis model. Tumor area, Sirius red-positive area, HABP-positive area, and *Has2* mRNA expression in mice fed LFD or HFD, with spleen-injected Pan02 cells. (n = 6)
Data are presented as mean ± SEM. Statistical significance was calculated with Student's 2-tailed t test or one-way analysis of variance followed by Tukey post hoc test. P values are indicated ($^{*}P < 0.05$ or $^{**}P < 0.01$).
(F) Representative fluorescence images of LratCre-tdTomato with immunofluorescence staining for α-SMA (left) and Desmin (right).
(G) Representative microscopic fluorescence images of LratCre-tdTomato and immunofluorescence staining of CD44 in liver tissues with metastatic tumor burden. Scale bar: 200 μm

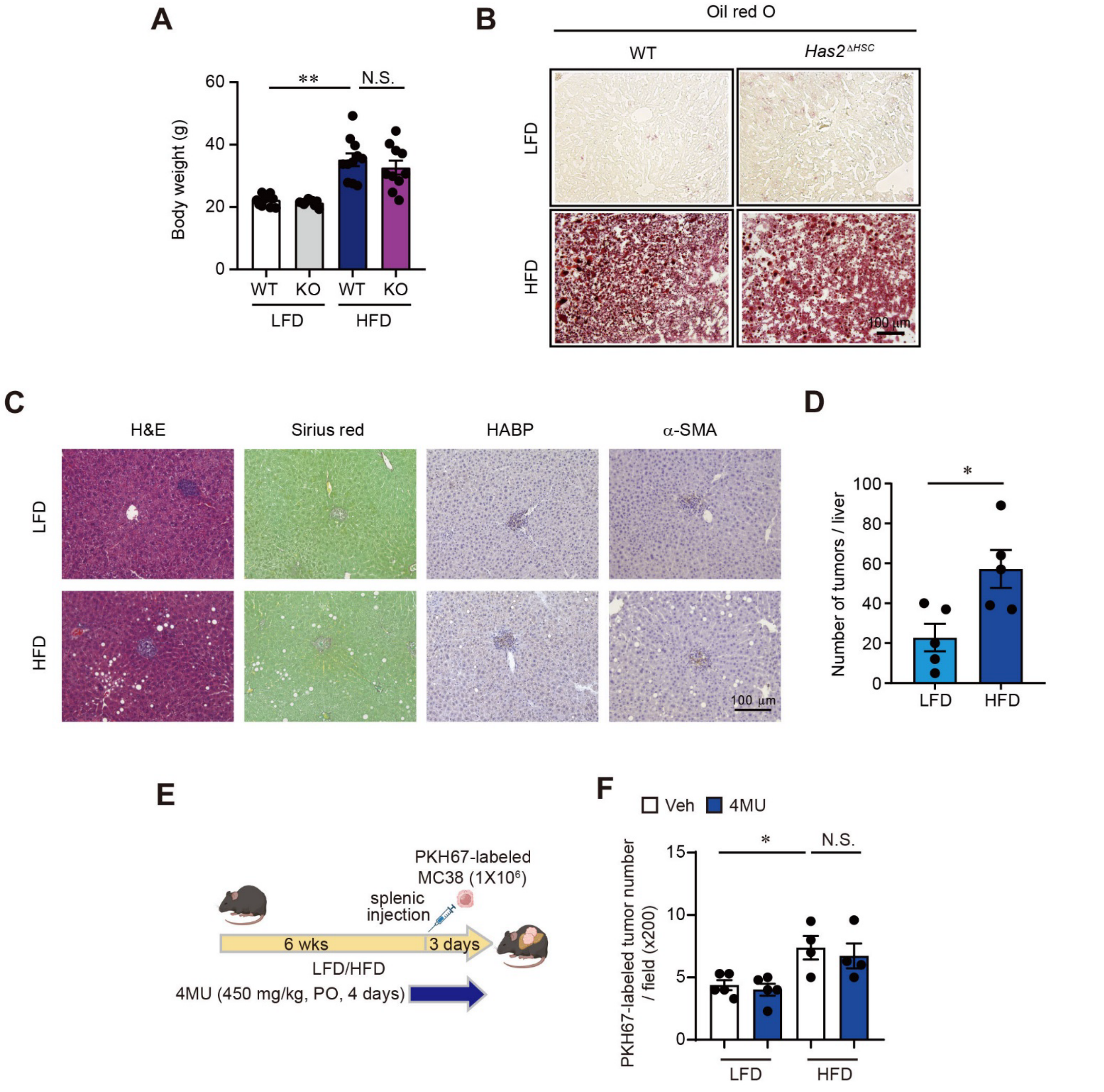

Supplemental Figure 2. Suppression of metastatic liver tumor growth through hepatic stellate cell-specific *Has2* deficiency in MASLD, independent of early engraftment.

(A) Analysis of body weight. Wild-type (WT) and *Has2*$^{\Delta HSC}$ mice were intrasplenically injected with MC38 cells after 6 weeks of either low-fat diet (LFD) or high-fat diet (HFD) feeding. Mice were maintained on their respective diets for an additional 2 weeks. (*n* = 10-12 per group)

(B) Representative images of oil red O staining of livers. Scale bar: 100 μm

(C-D) Measurement of early engraftment of metastatic tumor cells in the liver. Mice were fed either a LFD or HFD for 6 weeks, followed by spleen injection of MC38 cells. The experiment was concluded 3 days after tumor injection. (C) Representative images of H&E, Sirius red, HABP, and α-SMA staining are shown. Scale

bar: 100 μm (D) Number of tumors per liver. ($n = 5$)

(E) In vivo study design. Mice were fed either a LFD or HFD for 6 weeks. Starting one day before PKH67-labled MC38 spleen injection, 4-MU was administered orally at a dose of 450 mg/kg once daily for a total of 4 days.

(F) PKH-labeled tumor number. Liver sections were examined under fluorescent microscopy, and tumor foci were counted at 200× magnification. (n = 4-5)

Data are presented as mean ± SEM. Statistical significance was calculated with one-way analysis of variance followed by Tukey post hoc test (A, F) and Student's *t*-test (D). *P* values are indicated ($^{*}P < 0.05$ or $^{**}P < 0.01$). HFD, high-fat diet; KO, knockout; LFD, low-fat diet; N.S., not significant; WT, wild-type.

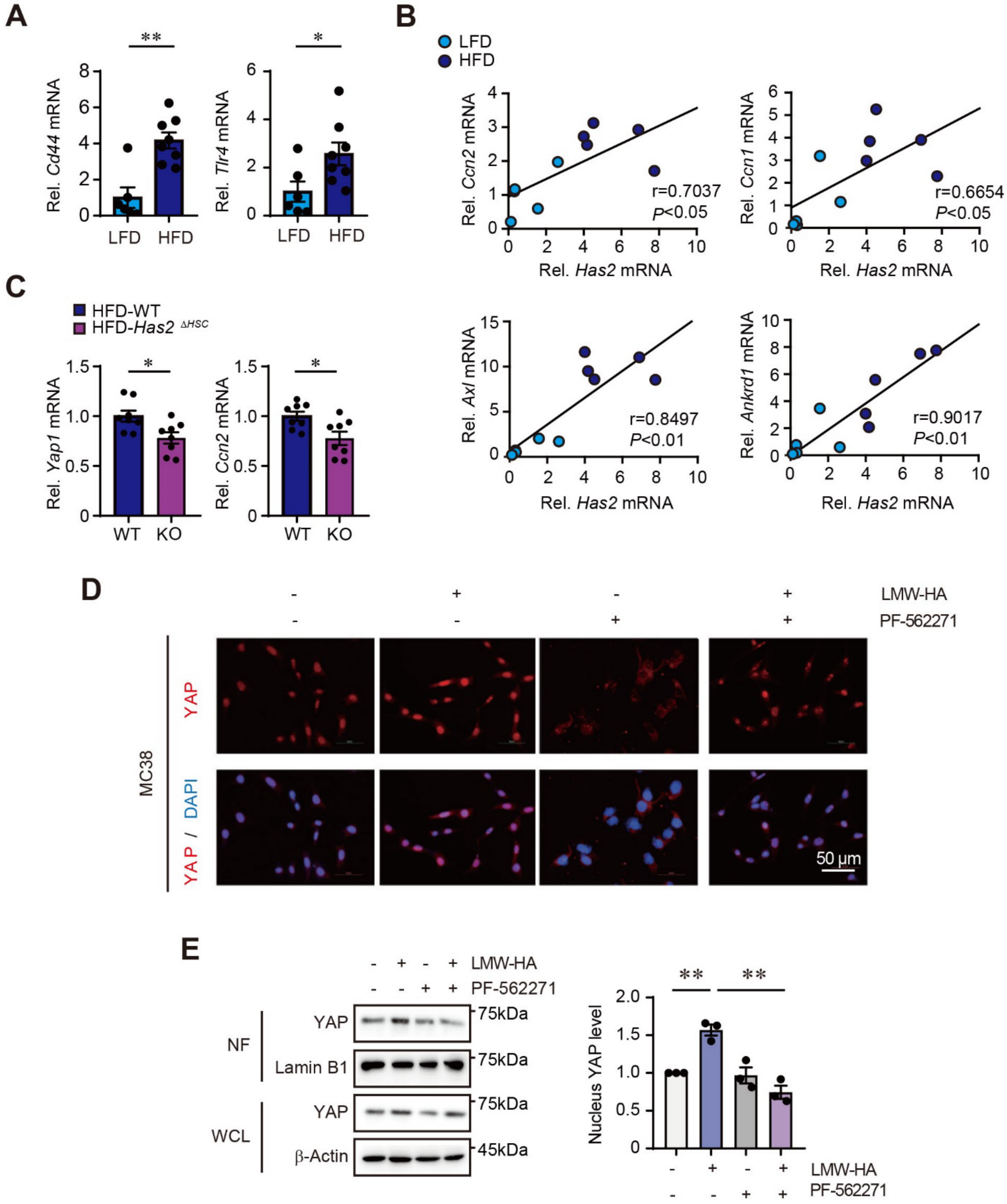

Supplemental Figure 3. HAS2 and LMW-HA regulate YAP activation.

(A) *Cd44* and *Tlr4* mRNA expression in tumors from mice fed either a low-fat diet (LFD) or a high-fat diet (HFD). (*n* = 6-8 per group)

(B) Quantitative reverse-transcription polymerase chain reaction assays were conducted to measure *Ccn2*, *Ccn1*, *Axl,* and *Ankrd1* mRNA expression in tumors. The Pearson correlation coefficient (r) was calculated. (*n* = 5)

(C) Effect of hepatic stellate cell (HSC)-specific *Has2* deletion on the mRNA expression of *Yap1* and *Ccn2* in tumors from HFD-fed wild-type (WT) mice or knockout (KO) mice. ($n = 8$)
(D) Immunocytochemical analysis of YAP. MC38 cells, serum-starved prior to treatment, were exposed to 4 μM PF-562271 for 30 minutes and subsequently incubated with 0.5 μg/mL HA for 2 hours.
(E) Western blot analysis of YAP in nuclear fractions (NF) and whole-cell lysates (WCL). MC38 cells were treated with to 4 μM PF-562271 for 30 minutes, followed by 0.5 μg/mL HA for 12 hours. The left panel shows representative blot images, while the right panel presents quantification of nuclear YAP levels. ($n = 3$)
Data are presented as mean ± SEM. Statistical significance was calculated with Student's *t*-test (A, C) and one-way analysis of variance followed by Tukey post hoc test (E). *P* values are indicated ($^{*}P < 0.05$ or $^{**}P < 0.01$).

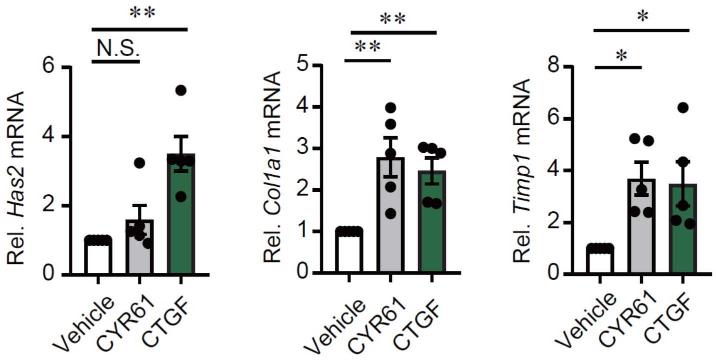

Supplemental Figure 4. CTGF, but not CYR61, increases *Has2* mRNA expression.

Quantitative reverse-transcription polymerase chain reaction assays for *Has2*, *Col1a1* and *Timp1* mRNA expression levels in mouse primary hepatic stellate cells (HSCs). The primary HSCs were treated with 10 ng/ml of recombinant cysteine-rich, angiogenic inducer 61 (CYR61) or 20 ng/ml of recombinant connective tissue growth factor (CTGF) protein for 24 hours. (n = 5)

Data are presented as mean ± SEM. Statistical significance was calculated with one-way analysis of variance followed by Tukey post hoc test. P values are indicated ($^{*}P < 0.05$ or $^{**}P < 0.01$). N.S., not significant

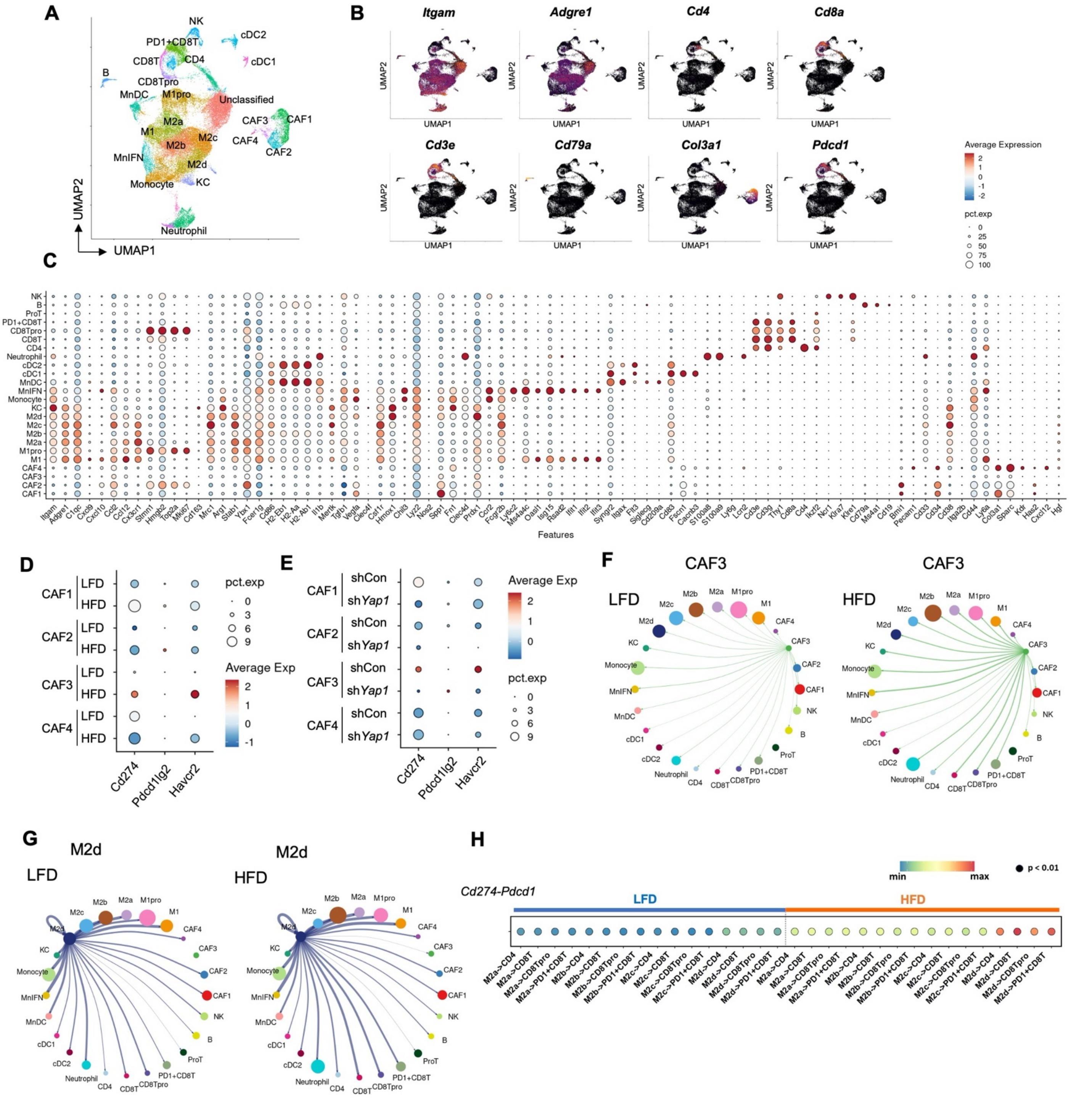

Supplemental Figure 5. CAF-derived hyaluronan synthase 2 and cancer-derived YAP contribute to the prometastatic immune tumor microenvironment in steatotic liver. (A) Determination of tumor-infiltrating immune cell populations. UMAP of single-cell RNA-seq analyses from 46,577 CAF and immune cells showing 25 clusters determined by integrated analysis, colored by cluster. Cells were isolated from metastatic liver tumors of mice fed a low-fat diet (LFD) or a high-fat diet (HFD) (n = 3/group). **(B)** Expression levels of key cluster-identification genes. **(C)** Dot plot for key cluster signatures (columns) by specific subpopulations (rows). Dot size represents the cell fraction within each subpopulation. Fill color indicates average expression. **(D,E)** Dot plot for expression of *Cd274*, *Pdcd1lg2*, and *Havcr2* genes (columns) by specific CAF subpopulations (rows). Dot size represents the cell fraction within the CAF subpopulations. Fill color indicates average expression (exp.). (D) Cells from tumors of LFD-fed and HFD-fed mice; (E) cells from control and *Yap1*-

silenced tumors of HFD-fed mice. Con, control; sh, short hairpin. **(F-H)** CellChat receptor-ligand analysis of the predicted intercellular communication networks for cells from metastatic liver tumors of LFD-fed and HFD-fed mice. Different cell subpopulations are represented by different colors within the circle plot. Arrows are proportional to the interaction strength between CAF3 (F) or M2d (G) and other cells, while node size is relative to the number of cells within that population. (H) The PD-L1 (*Cd274*) and PD-1 interaction strengths between M2 macrophage subpopulations and T cell subpopulations were shown.

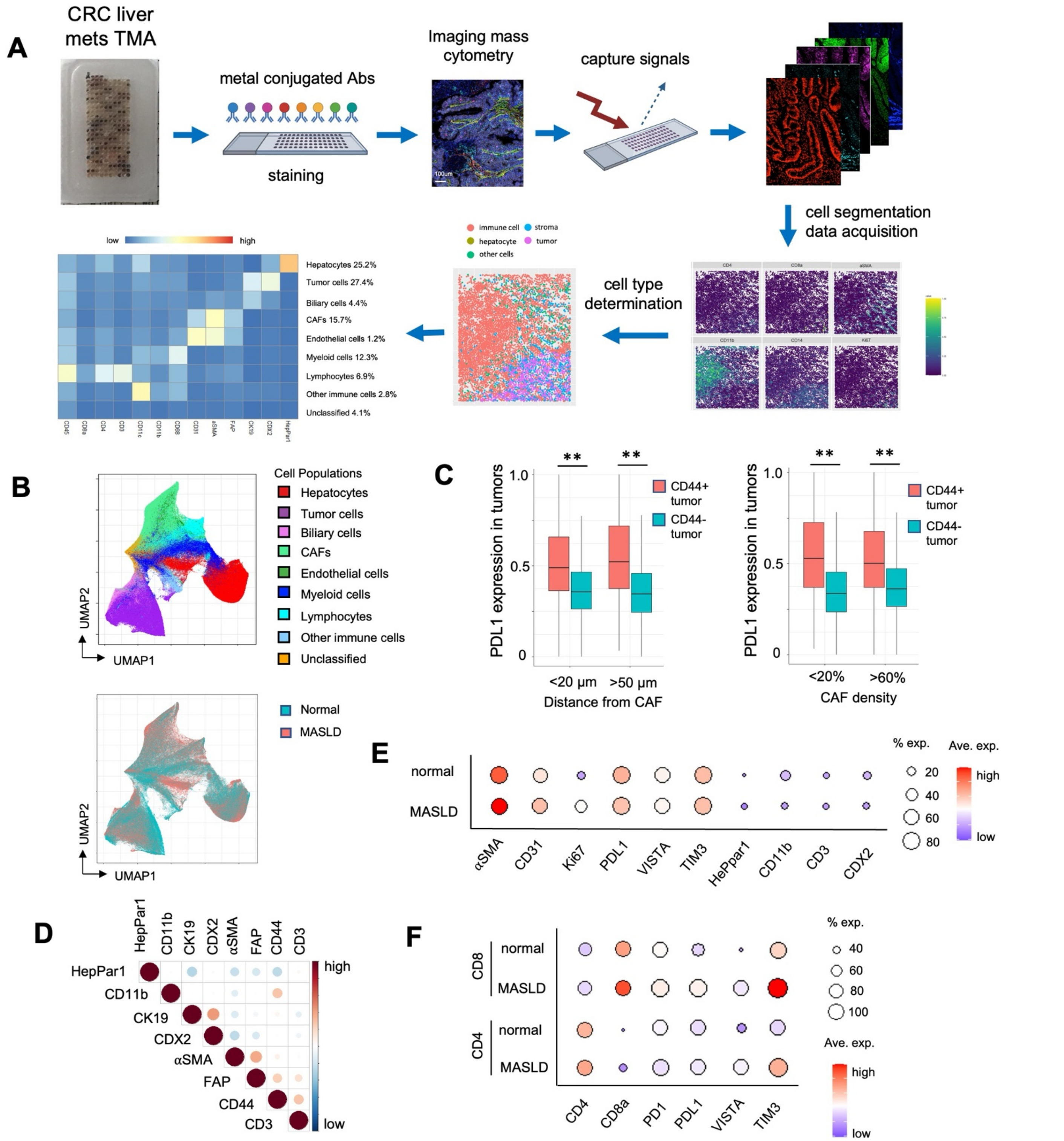

Supplemental Figure 6. Increased CAF infiltration and immunosuppressive tumor-associated macrophage and T cell phenotypes in patients with CRC liver metastasis with steatotic liver. (A) An image of the tissue microarray (TMA) containing tissues from colorectal cancer (CRC) liver metastasis with metabolic dysfunction-associated steatotic liver disease (MASLD) (n = 13) and without MASLD (n = 17), and the standard workflow for imaging mass cytometry (IMC). Heat map of cell populations determined by key phenotyping molecules from IMC analysis from 350,979 cells. **(B)** UMAP from **(A)** showing nine clusters determined by integrated analysis, colored by cluster. UMAP colored by MASLD condition. **(C)** Spatial analysis of IMC to evaluate the relationship between cancer cell PD-L1 expression and (left) the cancer cell's distance from cancer-associated fibroblasts (CAFs) or (right) the cancer cell's density of CAFs. Orange, CD44-

positive cancer; blue, CD44-negative cancer. **(D)** Dot plot for coexpression of CD44 and key phenotyping molecules. SMA, smooth muscle actin. **(E)** Dot plot for expression of α-SMA, CD31, and key phenotyping and immunomodulatory molecules (columns) by CAF populations from patients with or without MASLD (rows). Dot size: the cell fraction within each cell population. Fill color indicates average expression (ave. exp.). **(F)** Dot plot for expression of key phenotyping and immunomodulatory molecules (columns) by T cell subpopulations in patients with or without MASLD (rows). Dot size: the cell fraction within each cell population. Fill color indicates average expression. Significance determined by generalized linear models. *P* values are indicated ($^{**}P < 0.01$).

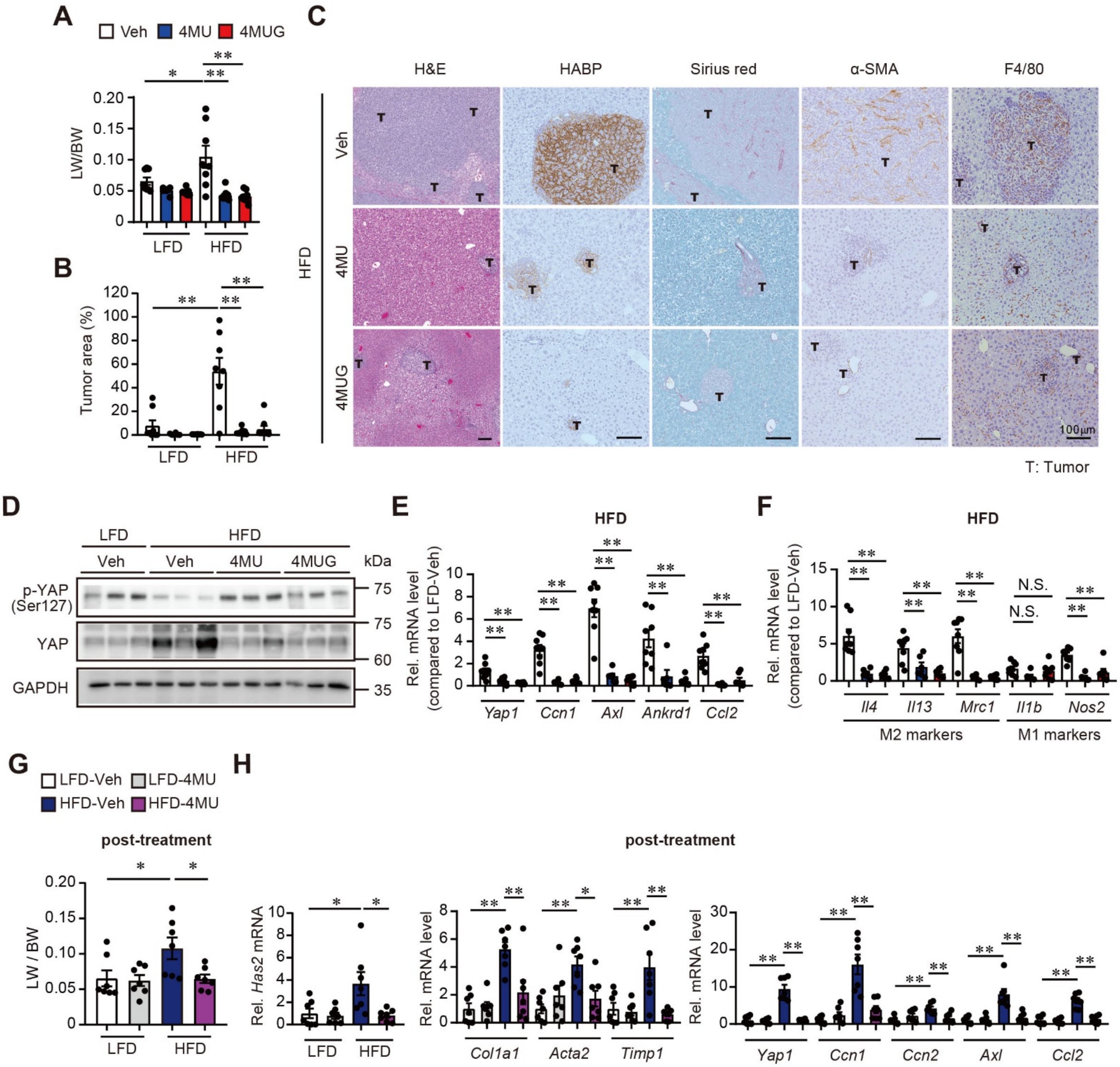

Supplemental Figure 7. Inhibition of hyaluronic acid synthesis alleviates metastatic liver tumor growth and CAF activation in the metabolic dysfunction-associated steatotic liver disease.

(A) Analysis of liver weight-to-body weight ratio (LW/BW) in the preventive intervention model. 4-methylumbelliferone (4-MU) was administered orally at a dose of 450 mg/kg, five times per week over a four-week period. Concurrently, 4-methylumbelliferyl glucuronide (4-MUG) was supplied in the drinking water at a concentration of 2 mg/mL for the same duration. (n = 6-9 per group) LFD, low-fat diet; HFD, high-fat diet; Veh, vehicle. **(B)** Assessment of tumor area (%). **(C)** Representative images of H&E, HABP, Sirius red, α-SMA, and F4/80 staining. T, tumor. Scale bar: 100 μm. **(D)** Western blot analysis of yes-associated protein (YAP) and phosphorylated YAP ($pSer^{127}$-YAP). **(E)** Measurement of mRNA expression levels for Hippo signaling-related genes in HFD-fed mice treated with the respective drugs. (n = 6-8 per group) **(F)** Quantitative reverse-

transcription polymerase chain reaction (qRT-PCR) assays of mRNA expression for M1 macrophage markers (*Il1b*, *Nos2*) and M2 macrophage markers (*Il4*, *Il13*, *Mrc1*) in tumors of HFD-fed mice treated with vehicle, 4-MU, or 4-MUG. **(G)** Evaluation of the LW/BW. Mice received 4-MU orally at a dose of 450 mg/kg daily, beginning one-week post-tumor inoculation and continuing for three additional weeks. (n = 7 per group) **(H)** Assessment of mRNA expression levels for *Has2* (left), *Col1a1, Acta2, Timp1* (middle), and *Yap1* and its target genes (right) in tumors from mice treated with 4-MU. Data are presented as mean ± SEM. Statistical significance was calculated with one-way analysis of variance followed by Tukey post hoc test. P values are indicated ($^{*}P < 0.05$ or $^{**}P < 0.01$). N.S., not significant

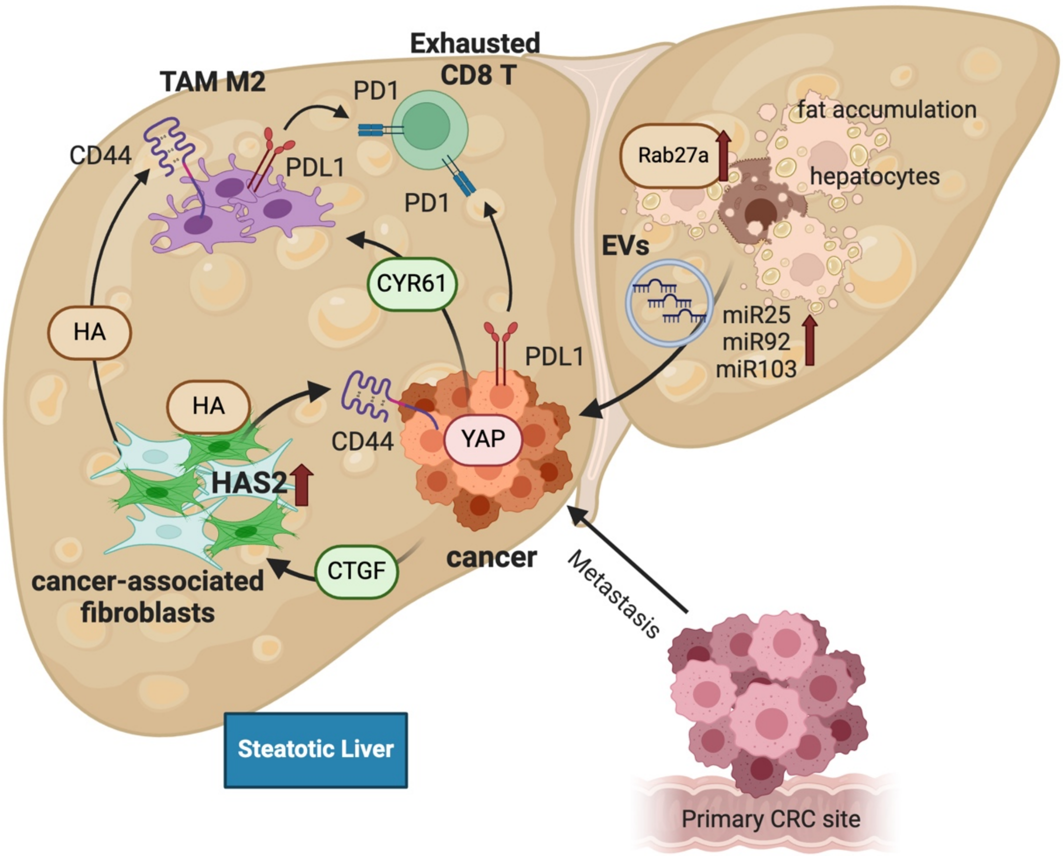

Supplemental Figure 8. Unified proposed mechanisms of enhanced liver metastasis in steatotic livers. Steatotic hepatocytes produce EVs. The EV-miRNAs create a pre- and pro-metastatic niche by increasing YAP activity in cancer cells and immunosuppressing microenvironment. Activated YAP produces CYR61 and CTGF from metastatic cancers. Tumor-derived CYR61 recruits and polarizes M2 macrophages, creating immunosuppressive TME with PD1 expressing exhausted CD8 T cells. On the other hand, tumor-derived CTGF stimulates intratumoral HSCs/CAFs to create fibrotic TME by upregulating HAS2 expression and HA production. HA, particularly LMW-HA, stimulates CD44 in tumors, further augmenting YAP activation to grow cancer. HA also affects M2 polarization and infiltration through CD44. PD-L1-expressing cancer cells and M2 macrophages then inhibit CD8 T cells through PD-1, further enhancing immunosuppressive TME and augmenting tumor growth. CAF, cancer-associated fibroblast; CTGF, connective tissue growth factor; CYR61, cysteine-rich angiogenic inducer 61; EV, extracellular vesicle; HA, hyaluronic acid; HAS2, hyaluronan synthase 2; HSC, hepatic stellate cell; LMW, low-molecular weight; TME, tumor microenvironment; YAP, yes-associated protein
