# Supplementary figures and images for: Metastatic tumor growth in steatotic liver is promoted by HAS2-mediated fibrotic tumor microenvironment
Source: J Clin Invest. 2025 Feb 13;135(7):e180802. doi: 10.1172/JCI180802 (PMC11957696; doi:10.1172/JCI180802)

Original western blots

Figure S3E

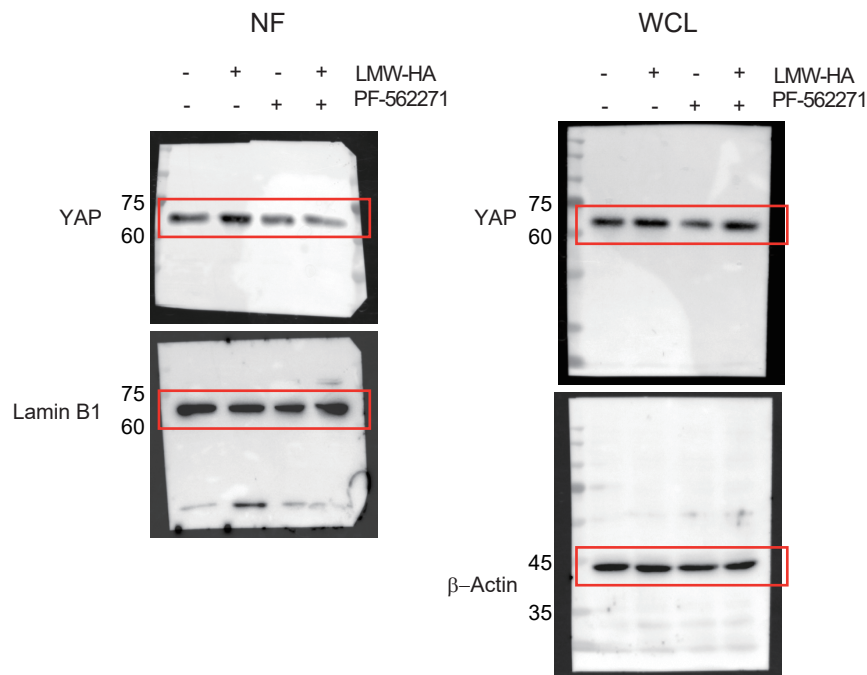

Figure 7F

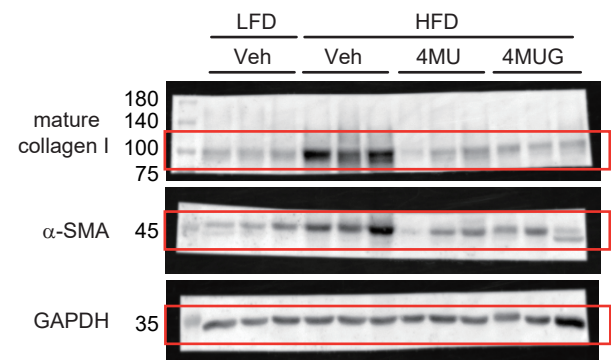

Figure S7D

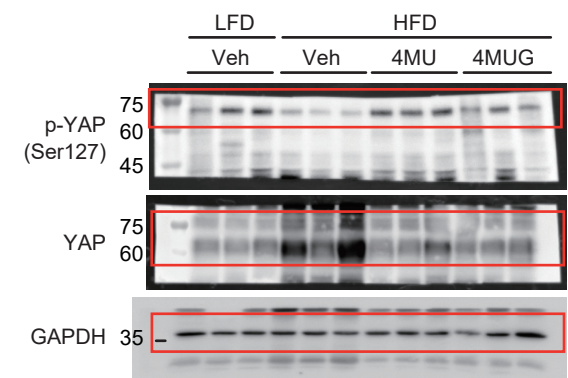

Supplement: Unedited blot and gel images [file jci-135-180802-s029.pdf]
